# Quantitative Cell Cycle Analysis Based on an Endogenous All-in-One Reporter for Cell Tracking and Classification

**DOI:** 10.1016/j.celrep.2017.05.022

**Published:** 2017-05-30

**Authors:** Thomas Zerjatke, Igor A. Gak, Dilyana Kirova, Markus Fuhrmann, Katrin Daniel, Magdalena Gonciarz, Doris Müller, Ingmar Glauche, Jörg Mansfeld

**Affiliations:** 1 Cell Cycle, Biotechnology Center, Technische Universität Dresden, 01307 Dresden, Germany; 2 Institute for Medical Informatics and Biometry, Carl Gustav Carus Faculty of Medicine, Technische Universität Dresden, 01307 Dresden, Germany

**Keywords:** cell cycle, cell fate decisions, cyclin oscillations, cyclin D1, p21, G1 phase regulation, quiescence, cell cycle reporter, quantitative single cell imaging, automated image analysis

## Abstract

Cell cycle kinetics are crucial to cell fate decisions. Although live imaging has provided extensive insights into this relationship at the single-cell level, the limited number of fluorescent markers that can be used in a single experiment has hindered efforts to link the dynamics of individual proteins responsible for decision making directly to cell cycle progression. Here, we present fluorescently tagged endogenous proliferating cell nuclear antigen (PCNA) as an all-in-one cell cycle reporter that allows simultaneous analysis of cell cycle progression, including the transition into quiescence, and the dynamics of individual fate determinants. We also provide an image analysis pipeline for automated segmentation, tracking, and classification of all cell cycle phases. Combining the all-in-one reporter with labeled endogenous cyclin D1 and p21 as prime examples of cell-cycle-regulated fate determinants, we show how cell cycle and quantitative protein dynamics can be simultaneously extracted to gain insights into G1 phase regulation and responses to perturbations.

## Highlights

• Endogenous mRuby-PCNA as an all-in-one cell cycle reporter
• Automated image analysis pipeline to segment, track, and classify based on PCNA
• Quantitative analysis of cyclin oscillations during a complete cell cycle
• Cyclin D1 maintains G1 phase and prevents the transition into quiescence

## Graphical Abstract

**Figure:**
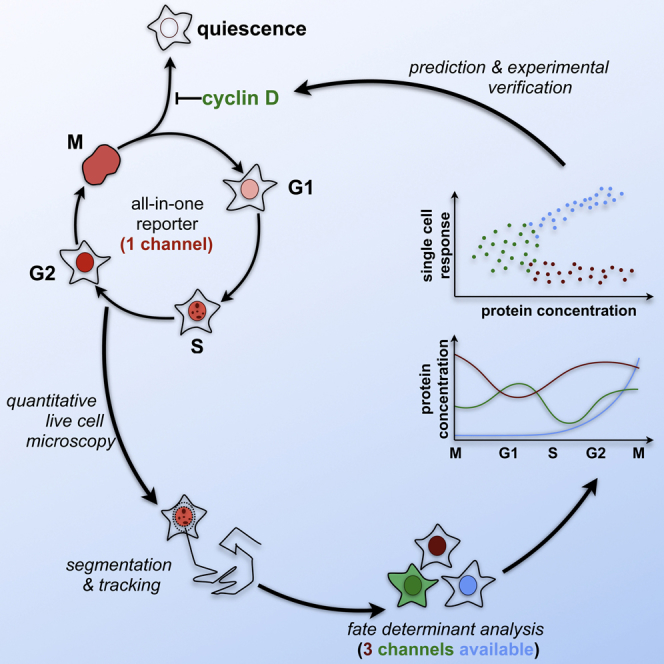

## Teaser

Zerjatke et al. present endogenously tagged PCNA as an all-in-one cell cycle reporter for living cells to classify all cell cycle phases and quiescence using a single fluorescent channel. Visualizing endogenous cyclin D1 in living cells, they show that cyclin D1 maintains G1 phase and prevents the transition into quiescence.

## Introduction

Cell fate decisions, such as reprogramming, differentiation, and cell cycle exit, are tightly linked to cell cycle kinetics. Moreover, the responsiveness of cells to internal and external stimuli varies depending on cell cycle stage. For instance, the availability of growth factors promoting proliferation over quiescence is sensed in late G2 phase and G1 phase, but not during S phase (Pardee, 1974, Spencer et al., 2013, Stacey, 2003, Zetterberg and Larsson, 1985). Similarly, cells respond in a cell-cycle-dependent manner to differentiation cues (Pauklin and Vallier, 2013) and DNA damage (Chandler and Peters, 2013, Soufi and Dalton, 2016). Hence, the time cells spend in individual cell cycle phases directly reflects the exposure to such stimuli and consequently their fate decision.

Throughout the cell cycle, spatiotemporal changes in the distribution of activators (e.g., cyclins) and inhibitors of cyclin-dependent kinases (CDKs) function to determine the length of individual cell cycle phases (Morgan, 2006). Indeed, this represents a paradigm for how the dynamic regulation of protein levels can regulate cellular decisions. The levels and spatiotemporal localization of cyclin D1 (CCND1) and the CDK inhibitor p21 (CDKN1A), for instance, have been suggested as crucial determinants of several cell fate decisions—indirectly by determining the length of G1 phase (Jiang et al., 1993, Musgrove et al., 1994, Resnitzky et al., 1994) and directly by regulating the choice between proliferation and quiescence at the recently proposed “maternal restriction point” (Chen et al., 2013, Spencer et al., 2013). However, to fully understand how cell cycle kinetics influences cell fate decisions, it will be necessary to gain a similar understanding of the spatiotemporal protein dynamics of cell fate determinants, such as pluripotency factors and differentiation factors, and to obtain detailed correlations with cell cycle kinetics in individual cells.

Long-term imaging of single cells, made possible by the remarkable advances in microscopy and image analysis techniques in recent years, has led us to re-evaluate long-standing models of cellular decision making (Barr et al., 2016, Chen et al., 2013, Guo et al., 2014, Spencer et al., 2013). However, gaining quantitative insights into cell cycle and protein dynamics within the same cell remains a major bottleneck. Current approaches to define cell cycle kinetics, including FUCCI-based reporters, typically rely on overexpressed reporters containing destruction degrons that are targeted for proteasomal degradation in a cell-cycle-dependent manner (Araujo et al., 2016, Bajar et al., 2016, Oki et al., 2014, Sakaue-Sawano et al., 2008). Depending on the reporter and the need for a further marker for segmentation, up to four channels (blue, yellow, red, and infrared; Bajar et al., 2016) are utilized to identify a cell and determine its precise cell cycle position. This limits concurrent analysis of multiple cell fate determinants, such as cyclin D1 and p21, within the same living cell and thus complicates investigation of causal relationships during decision making.

To resolve this issue, we present endogenous proliferating cell nuclear antigen (PCNA) as an all-in-one cell cycle reporter for live single-cell imaging that, unlike the aforementioned cell cycle reporters, only requires a single fluorescent marker to faithfully assign all cell cycle phases, including the transition into quiescence. We provide an accompanying image analysis pipeline for segmentation, tracking, and classification of single cells on the basis of PCNA abundance and localization, removing the need for a second marker, such as fluorescently tagged histones for automated segmentation, at least in proliferating cells. We demonstrate the simultaneous measurement of cell cycle and protein dynamics from single cells, providing a quantitative description of cyclin oscillations throughout the cell cycle. Our data suggest that neither cyclin D1 levels nor localization are general determinants of G1 phase length in unperturbed conditions. Instead, we find that cyclin D1 is required to keep cells in a proliferative mode and prevent the transition into quiescence. Finally, by visualizing the behavior of endogenous cyclin D1 and p21 following DNA damage, we illustrate how endogenous PCNA as an all-in-one cell cycle reporter can be used to study protein dynamics of multiple cell fate determinants in a perturbation-dependent manner within the same living cell. The endogenous reporters and the methodology described here represent not only a valuable resource to shed light on the decision making of individual cultured cells but also provide a framework for the simultaneous analysis of cell cycle and protein dynamics in more complex models.

## Results

### Dynamic Expression of Endogenously Tagged PCNA

**Figure 1:**
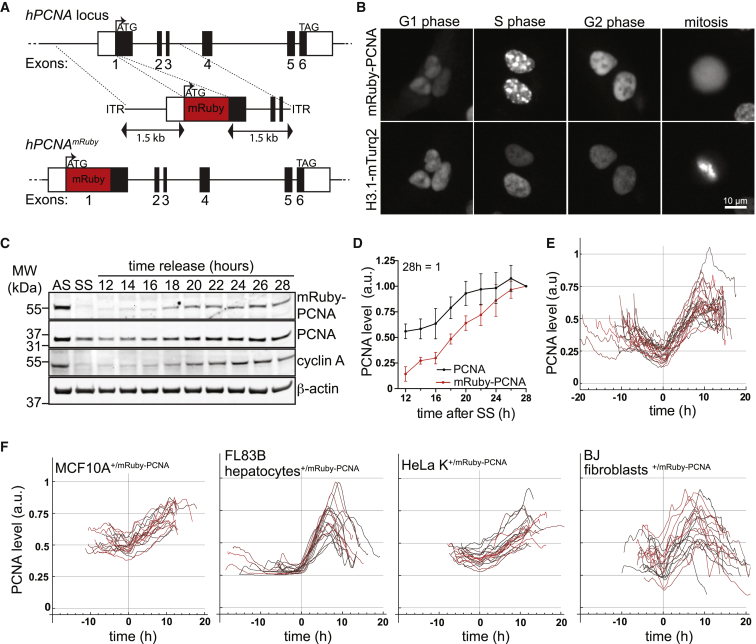
Dynamic Expression of Endogenous mRuby-PCNA (A) N-terminal targeting of endogenous PCNA with mRuby. (B) Cell cycle phase-dependent localization of endogenous mRuby-PCNA and histone 3.1-mTurquoise2. (C) Western blot analysis of a release from 24 hr serum starvation (SS), showing that untagged and tagged PCNA have similar expression kinetics; AS, asynchronously growing cells. Note that PCNA and mRuby-PCNA blots were imaged at different intensities to better illustrate the similar increase in PCNA expression. (D) Quantification of data shown in (C) represented as mean ± SEM from four independent experiments. (E) Single-cell tracks aligned to the beginning of S phase (t = 0 hr; see methodology), showing mRuby-PCNA levels during a complete cell cycle. (F) Single cell tracks as in (E), showing that the dynamic behavior of mRuby-PCNA is conserved in non-transformed and transformed human and murine cells. See also Figure S1.

Overexpression of PCNA fused at the N terminus to a fluorescent protein is widely used to label cells in S phase based on the presence of replication foci (Barr et al., 2016, Leonhardt et al., 2000, Leung et al., 2011, Piwko et al., 2010). *PCNA* expression is tightly coupled to proliferation peaking in G1/S (Santos et al., 2015) and decreasing upon cell cycle exit (Buttitta et al., 2010, Thacker et al., 2003). Thus, we reasoned that it might be possible to extend the utility of PCNA as a cell cycle reporter beyond S phase alone. To create an endogenously expressed *PCNA* reporter, we inserted the gene encoding the fluorescent protein mRuby in frame with the first exon into one allele of the *PCNA* locus by recombinant adeno-associated virus-mediated (rAAV) homologous recombination in non-transformed human retinal pigment epithelial cells (hTERT RPE-1) (Figure 1A). Endogenous mRuby-PCNA was expressed at a lower level than untagged PCNA (Figure S1A) but localized to the nucleus in interphase and was present in replication foci during S phase as expected (Figure 1B; Leonhardt et al., 2000). To ensure that the protein dynamics of mRuby-PCNA recapitulate untagged PCNA, we synchronized cells in G0 by serum withdrawal for 24 hr and monitored the expression from both alleles after addition of serum. Quantitative western blot analysis indicated similar expression kinetics of the tagged and untagged alleles, suggesting that mRuby-PCNA faithfully recapitulates this aspect of endogenous PCNA regulation (Figures 1C and 1D).

To establish an independent reference for segmentation and tracking of mRuby-PCNA-expressing cells, we inserted a gene encoding the fluorescent protein mTurquoise2 into the histone 3.1 locus (*HIST1H3E*) (Figures 1B and S1B). Analysis of mRuby-PCNA expression in the nucleus of proliferating cells from mitosis to mitosis (see Experimental Procedures) revealed that the expression level remained low for the first 8–10 hr after mitosis and then gradually increased for a further 10 hr until a plateau was reached at about 2-fold higher than the initial level (Figure 1E). Further, mRuby-PCNA behaved the same way in human primary BJ-foreskin fibroblasts, HeLa and mammary MCF10A cells, and in murine FL83B hepatocytes (Figures S1C and 1F). These data indicate that the behavior of endogenously tagged PCNA is conserved across primary, transformed, and non-transformed cells derived from various tissues and organisms. Given that PCNA is present in all proliferating cells, the endogenous mRuby-PCNA reporter could serve as a universal tool for the analysis of cell cycle and protein dynamics.

### Loss of mRuby-PCNA Is an Early Marker of Cell Cycle Exit and Quiescence

**Figure 2:**
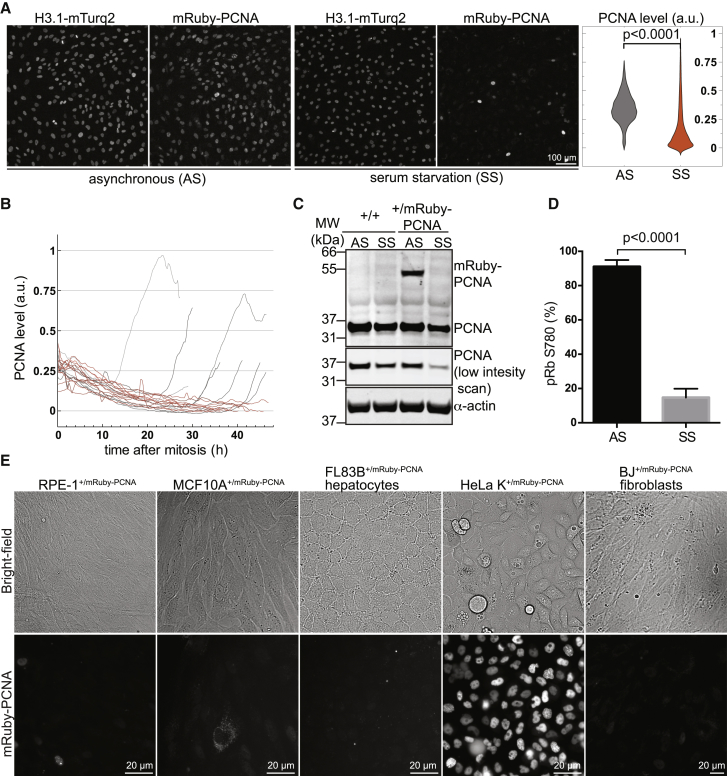
Loss of mRuby-PCNA Is an Early Marker of Cell Cycle Exit and Quiescence (A) Images and quantification of asynchronously growing (AS) or 48 hr SS RPE-1 cells expressing endogenous mRuby-PCNA and histone 3.1-mTurqouise2. (B) Single-cell tracks showing the decline of mRuby-PCNA levels during 48 hr of serum starvation. (C) Western blot analysis of cells treated as in (A), showing the decrease of untagged and tagged PCNA in response to 48 hr of serum starvation. (D) Quantification of pRb780 positivity in cells treated as in (A) represented as mean ± SEM from two independent experiments with greater than five technical replicates. (E) Images showing the decrease of mRuby-PCNA in non-transformed human and murine cells, but not in HeLa cells, in response to contact inhibition. See also Figure S1.

The presence or absence of PCNA is commonly used to distinguish proliferating cells from others that are quiescent or post-mitotic in fixed samples. To determine whether the loss of endogenously tagged PCNA is also indicative of cell cycle exit during live-cell imaging, we serum starved cells for 48 hr to induce quiescence. Indeed, compared to asynchronously growing cells, there was a significant and consistent reduction in nuclear mRuby-PCNA fluorescence in the majority of cells upon serum removal (Figure 2A). Single-cell analyses revealed that mRuby-PCNA expression declined gradually over a period of 48 hr (Figure 2B), and western blot and qPCR analyses revealed similar behavior for tagged and untagged PCNA (Figures 2C and S1D). A strong reduction in retinoblastoma (Rb) serine 780 phosphorylation, a marker of all cell cycle phases apart from quiescence (Narasimha et al., 2014), confirmed that, after 48 hr of serum starvation, the majority of cells exited the cell cycle (Figures 2D and S1E). Contact inhibition is an alternative and possibly more physiological approach to induce cell cycle exit and quiescence in cell culture. We therefore monitored the behavior of mRuby-PCNA in cells grown to confluence from the different mRuby-PCNA knockin cell lines. Primary and non-transformed cell types showed strongly reduced mRuby-PCNA levels after both serum starvation and contact inhibition, whereas no such reductions were observed in HeLa cells (Figures 2E and S1F). Together, these data indicate that the loss of mRuby-PCNA can be used as an early marker of cell cycle exit in different cell types and organisms.

### Robust Cell Cycle Classification Based on PCNA Expression and Localization

**Figure 3:**
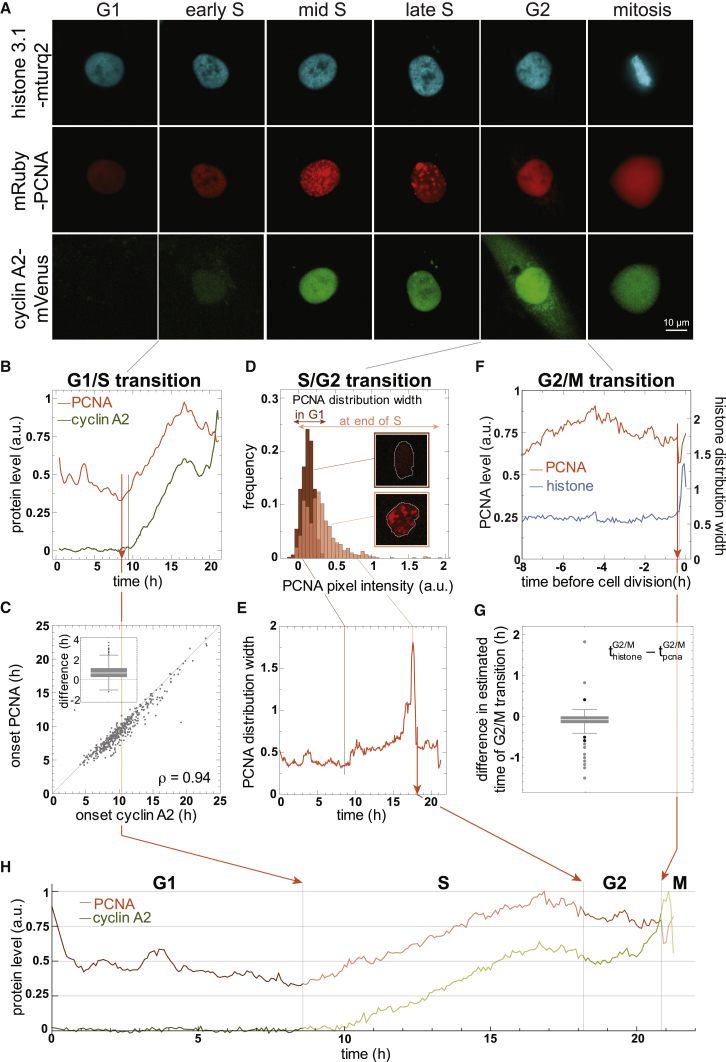
Robust Cell Cycle Classification Based on mRuby-PCNA (A) Cell cycle phase-dependent localization of endogenous mRuby-PCNA, histone 3.1-mTurquoise2, and cyclin A2-mVenus in living RPE-1 cells. (B and C) Representative single cell track (B) and scatterplot (C), illustrating the correlated onset of mRuby-PCNA and cyclin A2-mVenus expression. Note that mRuby-PCNA precedes cyclin A2-mVenus on average by 45 min (insert) and is used to define the beginning of S phase (n = 591). See also Movie S2. (D) Distribution of mRuby-PCNA intensity before (dark red) and at the end (light red) of S phase. (E) The distribution width of mRuby-PCNA intensity is indicative of the S/G2 transition. (F) The maximum distribution width of histone 3.1-mTurquoise2 indicative of mitosis coincides with a drop in mRuby-PCNA intensity due to redistribution of mRuby-PCNA to the cytoplasm after nuclear envelope breakdown. (G) Difference in estimated time of G2/M transition based on histone and PCNA levels as illustrated in (F). (H) Representative single-cell track with classification of cell cycle phases. See also Figure S2.

The dynamic behavior of PCNA foci during S phase previously enabled machine-learning-assisted automated classification of G1, S, and G2 phases during time-lapse microscopy (Held et al., 2010, Piwko et al., 2010). To precisely and reliably identify the onset of S phase, it is necessary to identify the first small replication foci. However, this is challenging in applications that require lower resolution microscopy, for instance during small molecule inhibitor screens or selective plane illumination microscopy (SPIM), which performs very well at low numerical apertures. We therefore explored whether increased mRuby-PCNA expression 8–10 hr after mitosis could serve as a marker for entry into S phase at low resolution (10× magnification). As an independent reference for S phase entry, we used both tagged endogenous cyclin A2-mVenus (Collin et al., 2013, Mansfeld et al., 2011) and the FUCCI reporter mAG-hGem, which accumulates at the G1/S transition due to anaphase-promoting complex/cyclosome (APC/C) inactivation (Sakaue-Sawano et al., 2008). Indeed, appearance of cyclin A2-mVenus and mAG-hGem correlated strongly with mRuby-PCNA expression (Figures 3A–3C and S2B; Movies S2 and S5), with PCNA expression preceding that of cyclin A2 and mAG-hGem by 45 and 16 min, respectively (Figures 3C and S2B, see inserts). Thus, increased PCNA expression provides a simple and robust measure for the onset of S phase in single-cell analyses.

To detect the end of S phase, we used the distribution width of mRuby-PCNA intensity as a proxy for PCNA granularity. In G1 and G2 phases, when replication foci are absent, uniform distribution of mRuby-PCNA across the nucleus results in a narrow distribution width of pixel intensities. In contrast, during S phase, the distribution width increases along with the formation of increasingly larger and thus brighter replication foci, leading to a maximum at the end of S phase that can be used to classify the S/G2 transition (Figures 3D and 3E). Mitosis is uniquely characterized by the significant drop in mRuby-PCNA levels at nuclear envelope breakdown and confirmed by the strong increase in the signal distribution width of histone 3.1 (Figures 3F and 3G). Taken together, our approach provides a framework for the robust and automated assignment of all cell cycle phases at low resolution based on the dynamic behavior of endogenous PCNA alone (Figures 3H and S2).

### mRuby-PCNA-Based Cell Segmentation, Tracking, and Classification

**Figure 4:**
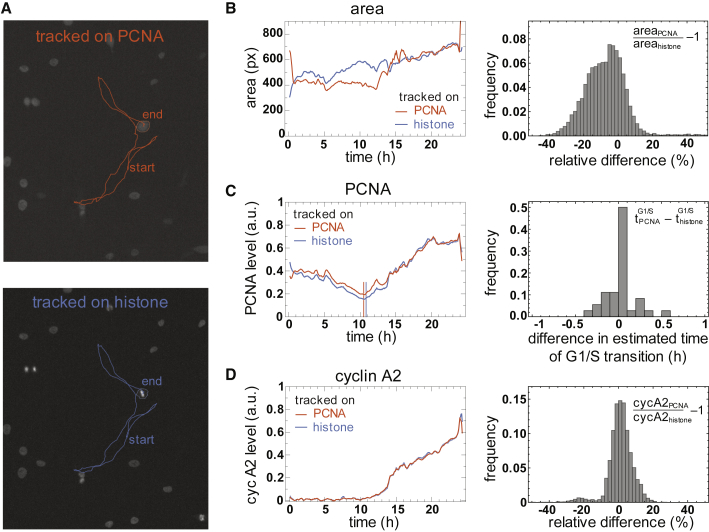
mRuby-PCNA as an All-in-One Cell Cycle Reporter (A) Representative single-cell trajectories for comparison of segmentation and tracking on histone 3.1-mTurquoise2 (blue) or mRuby-PCNA (red) images. (B–D) Representative time courses and quantified differences between the two segmentation approaches in area (B), mRuby-PCNA (C), and cyclin A2-mVenus levels (D). Though segmented areas can differ, especially for low levels of PCNA during G1 (B), the time point of G1/S transition is estimated very precisely with no deviation of >0.5 hr (C) and cyclin A2-mVenus levels can be robustly quantified with <10% deviation for almost 90% of the time points with positive signal (D; n = 36 cells). See also Figure S2 and Movie S1.

In addition to classification of cell cycle stages, the relatively strong expression of endogenous PCNA raises the possibility of using mRuby-PCNA to segment the nuclei of proliferating cells. Comparison of histone-based segmentation with an mRuby-PCNA-based approach revealed that single cells could be reliably followed on either of the two channels, leading to the same cell tracks (Figure 4A) during live-cell imaging. Comparison of nuclear sizes revealed only a minor difference between PCNA and histone strategies (Figure 4B), mainly during G1, when PCNA levels are lower. To compensate for this effect, we introduced an additional local re-segmentation step after tracking (see Supplemental Experimental Procedures). Minor differences remained after nuclear envelope breakdown, when histone 3.1-mTurquoise2 only marks the condensed chromosomes whereas PCNA becomes distributed throughout the cell. Consequently, mRuby-PCNA and cyclin A2-mVenus dynamics in the nucleus (Figures 4C and 4D) were almost indistinguishable between PCNA and histone segmentation. Finally, both segmentation methods produced almost identical predictions of the G1/S transition. Thus, endogenous mRuby-PCNA can serve as an all-in-one cell cycle reporter in time-lapse analysis of proliferating cells.

### Quantitative Determination of Endogenous Protein Dynamics in Single Cells

An enticing feature of mRuby-PCNA as an all-in-one reporter for segmentation, tracking, and fluorescence extraction is the possibility of simultaneously monitoring the dynamic behavior of other fluorescently tagged proteins within the same cell. We therefore investigated this possibility by analyzing cyclin oscillations, a prime example of specific protein dynamics that span all cell cycle phases. Despite their discovery more than 30 years ago (Evans et al., 1983), endogenous cyclin oscillations have not yet been visualized in single living cells nor have their relative levels been quantitatively compared in a cell-cycle-dependent manner. We created RPE-1 cell lines expressing mRuby-PCNA together with one allele of endogenously tagged cyclin A2-mVenus, cyclin B1-mVenus, or cyclin D1-mVenus created by rAAV-mediated homologous recombination (Figure S3A). We have previously shown that endogenous cyclin A2-mVenus and cyclin B1-mVenus fusions are faithfully degraded during mitosis, suggesting that recognition and ubiquitylation by the APC/C is recapitulated by the fluorescently labeled fusion proteins (Collin et al., 2013, Mansfeld et al., 2011). To ensure that cell-cycle-dependent expression and accumulation of the fusion proteins were unperturbed, we monitored the expression of the tagged and untagged allele during release from a G1/S arrest for cyclins A2 and B1 (Figures S3B and S3C) or after a translational shutdown by cycloheximide in the case of cyclin D1 (Figure S3D). In all cases, the expression kinetics of both alleles was comparable and cyclin localization in the nucleus and cytoplasm was consistent with that expected for the corresponding cell cycle stage (Figure S3E). We noticed, however, that tagged cyclin D1 did not decrease as fast as the untagged allele in response to treatment with the translation inhibitor cycloheximide, indicating a prolonged half-life of the fusion protein. Furthermore, pull-downs with a camelid single-domain antibody (VHH) specific for GFP from asynchronous cells confirmed that all cyclin-mVenus fusions interacted with the corresponding CDK1, CDK2, and CDK4 (Figures S3F and S3G). Given that all tagged cyclins localized in a cell-cycle-dependent manner as previously reported (Figures 3A and S3E), C-terminal tagging of cyclins with mVenus most likely does not interfere with these aspects of cyclin regulation.

In principle, addition of the same fluorescent tag to all cyclins should allow quantitative comparison of cyclin behavior and enable correlative approaches to model cell cycle decisions. Because we only tagged one cyclin allele with mVenus, we employed quantitative western blot analysis based on linear near-infrared detection to compare levels of tagged and untagged cyclins in asynchronously growing cells (Figure S4A). Whereas the cyclin A2 fusion protein was expressed at similar levels to the untagged allele, cyclin B1-mVenus only accounted for 30% of total cyclin B1 whereas cyclin D1-mVenus represented 73% of total cyclin D1. Therefore, to estimate the total cyclin levels (tagged and untagged allele) from live-cell imaging, we accounted for differences in expression by correcting the fluorescence measurements based on quantitative western blot analysis of both alleles (Figures S4B and S4C). Furthermore, we standardized our fluorescence measurements using pure recombinant mVenus protein and exposure times that allow direct comparison of expressed cyclins (Figures S4D and S4E; Supplemental Experimental Procedures).

### Quantitative Analysis of Cyclin Oscillations throughout the Cell Cycle

**Figure 5:**
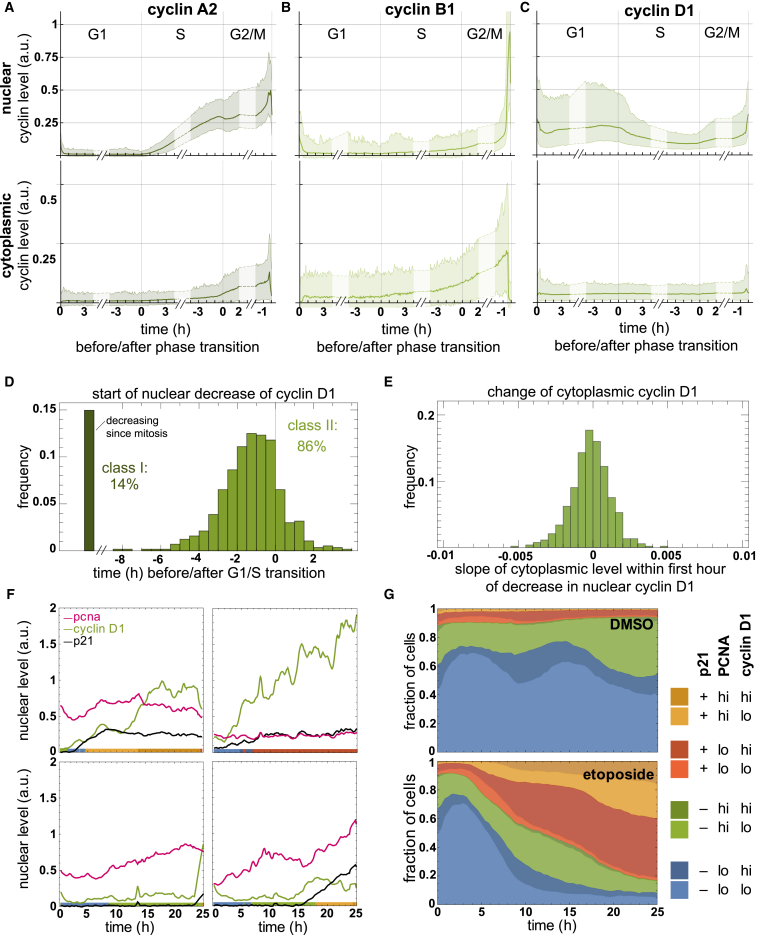
Quantitative Analysis of Cyclin Oscillations and Response of p21, Cyclin D1, and PCNA to DNA Damage (A–C) Quantitative cyclin dynamics of cyclins A2 (A), B1 (B), and D1 (C) in the nucleus or cytoplasm during a complete cell cycle. Average curves represent the combined levels of tagged and untagged cyclins A2, B1, and D1 according to quantitative western blot analysis (Figures S4A and S4B) and quantitative imaging (Figures S4D and S4E). Bands around the average curves show 95% of the values. Exemplary single-cell time courses are shown in Figure S5 and Movies S2, S3, and S4. (D and E) Histograms illustrating when cyclin D1 levels start decreasing in the nucleus (D) and the slope of cyclin D1-mVenus intensity in the cytoplasm at this time (E). The mean slope of almost 0 is in agreement with the constant levels of cytoplasmic cyclin D1 observed from mitosis to mitosis; n = 567 cells. (F) Representative single-cell tracks showing distinct dynamics of p21-mTurquoise2, mRuby-PCNA, and cyclin D1-mVenus in response to 1 μM etoposide. (G) Cumulative behavior of >2,500 single living cells treated as in (F). See also Figures S3, S4, and S6.

Taking advantage of our image analysis pipeline (Figure S2), we extracted relative nuclear and cytoplasmic intensities for cyclins during a complete cell cycle (mitosis to mitosis; Figures 5A–5C and S5A–S5C; Movies S1, S2, S3, S4, and S5). As expected, nuclear cyclin A2 expression began at the G1/S transition and often plateaued at the end of S phase. Starting from late S phase, cyclin A2 also accumulated in the cytoplasm, whereas nuclear cyclin A2 remained constant at high levels until shortly before mitosis (Figures 3B, 5A, and S5A). In contrast, cyclin B1 was expressed only in G2 phase in the cytoplasm and entered the nucleus about 20 min before mitosis, as previously described (Figures 5B and S5B; Gavet and Pines, 2010, Pines and Hunter, 1991). Notably, we also observed prominent oscillations of cyclin D1 from mitosis to mitosis (Figures 5C and S5C). First, nuclear cyclin D1 levels increased in early G1 phase, followed by a steep decrease that coincided with the beginning of S phase in most cells and a recovery toward late S and G2 phases (Figures 5C, 5D, and S5C). During the nuclear decrease, we observed no changes in the levels of cytoplasmic cyclin D1 (Figure 5E). Hence, cyclin D1 is either degraded in the nucleus or exported into the cytoplasm and subsequently degraded (Alao, 2007). By comparing how cyclin oscillations differed between single cells, we noticed a more heterogeneous behavior of cyclin D1 compared with cyclins A2 and B1, especially during G1 phase (compare bandwidth in Figures 5A–5C). Taken together, these data demonstrate how quantitative protein dynamics in the nucleus and cytoplasm can be extracted in a cell-cycle-dependent manner and how quantitative imaging can be employed for comparisons between different endogenous PCNA reporter lines.

### Parallel Determination of Distinct Protein Dynamics in Single Living Cells

Most cell fate decisions likely involve the dynamic interplay of more than one fate determinant. Therefore, the ability to extract single-cell protein dynamics for multiple factors in parallel would be of immense benefit to studies of cellular decision making and heterogeneity. As the mRuby-PCNA-based all-in-one reporter utilizes only a single wavelength for live-cell imaging, the dynamics of at least two additional proteins (tagged with yellow and blue fluorescent proteins) can be visualized and directly compared within the same cell. To demonstrate this feature, we focused on cyclin D1 and p21, which have been proposed as key determinants of cell cycle decisions but have not yet been monitored in parallel within the same cell in live-cell imaging experiments. For this, we tagged one allele of p21 with the fluorescent protein mTurquoise2 on the background of endogenous mRuby-PCNA and cyclin D1-mVenus (Figures S6A and S6B). p21 levels were generally very low or undetectable in unperturbed conditions but increased dramatically in most cells after treatment with the DNA-damaging reagent etoposide (Figures S6C and S6D). Next, we asked how p21, cyclin D1, and PCNA respond to extended etoposide treatment and whether the response at the single-cell level is similar or different to that of the entire population. The population analysis suggested that p21, cyclin D1, and PCNA levels generally increased by the end of the experiment (Figure S6C). However, by monitoring the behavior of individual cells, we uncovered substantial heterogeneity in the kinetics of the individual proteins during the manifestation of the endpoint phenotype (Figure 5F). A corresponding summary statistic is compiled in Figure 5G. Thus, our parallel measurement of multiple cell cycle regulators in individual cells reveals distinct heterogeneity, which was previously not accessible on the population level.

### Cyclin D1 Abundance Is a Poor Predictor of G1 Phase Length

**Figure 6:**
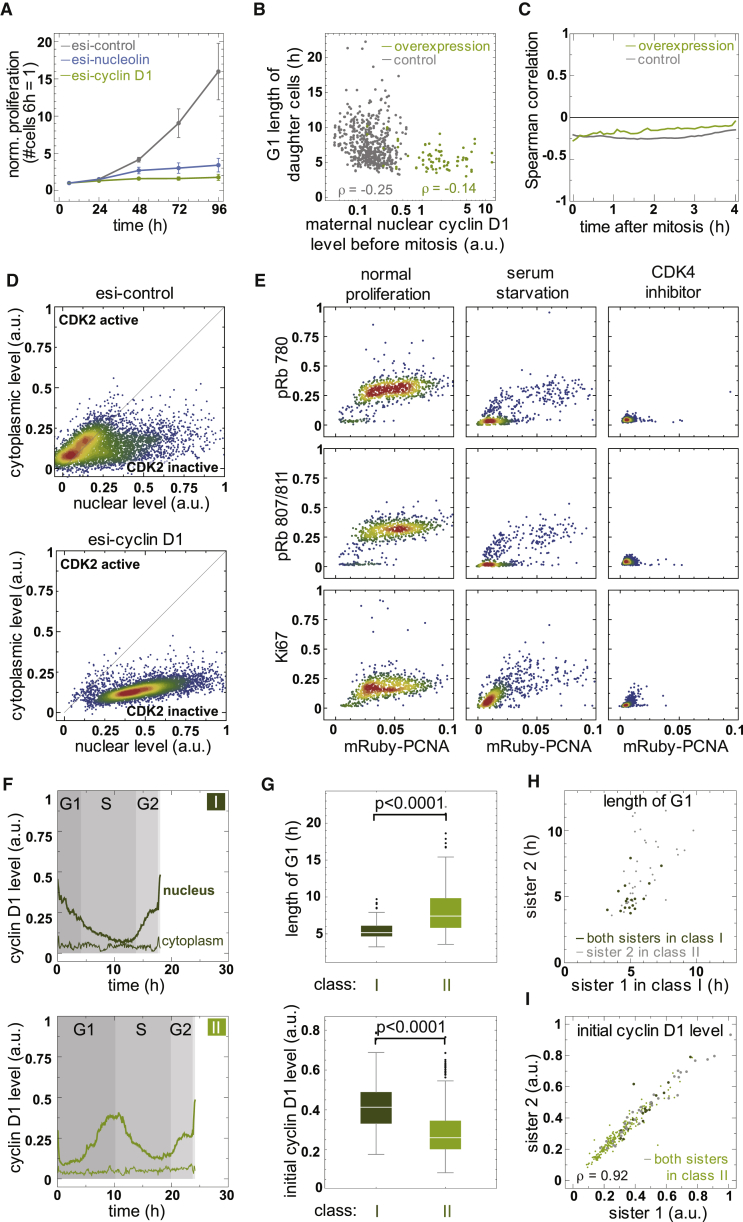
Cyclin D1 Maintains G1 Phase and Prevents the Transition into Quiescence (A) Normalized proliferation of RPE-1 cells treated with the indicated esi-RNAs represented as mean ± SEM from three independent experiments. (B) Scatterplot showing maternal cyclin D1 levels before mitosis in relation to G1 phase length in daughter cells of cyclin D1-mVenus- (gray) and cyclin D1-CDK4-mVenus (green)-expressing cells. Note that the expression of constitutively active cyclin D1-CDK4-mVenus does not shorten G1 phase beyond a minimum time that is also supported by endogenous levels of cyclin D1. (C) Correlation between expression level and G1 length of daughter cells derived from the experiment shown in (B). (D) Scatterplot showing nucleoplasmic and cytoplasmic levels of DHB-Venus in control and cyclin D1-depleted cells. Note nuclear DHB-Venus indicates inactive CDK2, a hallmark of CDK2^low^ cells. (E) Scatterplot showing pRb and Ki67 staining versus mRuby-PCNA levels in cells treated with the indicated esi-RNAs for 48 hr. (F) Representative single-cell tracks of nucleoplasmic and cytoplasmic cyclin D1 levels grouped into two classes according to cyclin D1 dynamics during G1 phase. (G) G1 phase length and initial nuclear level of cyclin D1 after mitosis in cells belonging to class I and II as defined in (F). (H) Scatterplot showing the relation of G1 phase length and classification as defined in (F) in sister cells. (I) Scatterplot illustrating the amount of maternally inherited cyclin D1 in between sister cells. Gray and dark green dots represent sister cells as defined in (H). Note that the initial level of cyclin D1 in sister cells is highly correlated independently of their class.

Moderate overexpression of cyclin D1 in cell lines (Jiang et al., 1993, Quelle et al., 1993, Resnitzky et al., 1994) has been reported to accelerate G1 phase and thus proliferation. Hence, cells that enter the cell cycle with elevated levels of cyclin D, as observed in many cancer cells, should progress to S phase faster because less additional proliferative input is required to inactivate Rb. The endogenous cyclin D1/PCNA reporter and associated methodology we present here prompted us to evaluate this prediction, especially in light of a new model of G1 phase regulation that challenges the long-standing paradigm (Bertoli and de Bruin, 2014, Narasimha et al., 2014). We first verified by semiquantitative PCR and proliferation analyses that cyclin D1 was the limiting D-type cyclin in RPE-1 cells and thus a suitable proxy for all D-type cyclins in this setting (Figures S5D and 6A). Next, we asked whether the amount of nuclear cyclin D1 before mitosis in the mother or during the first 4 hr after mitosis in daughter cells is a good predictor of G1 length. However, levels of cyclin D1 in maternal or daughter cells correlated only weakly with the length of G1 phase within the overall population, with mainly extremely low cyclin D1 expressing cells exhibiting longer G1 phases (Figures 6B and 6C). Because the level of cyclin D1 is not necessarily always synonymous with CDK4/6 activation, we expressed a constitutively active cyclin D1-CDK4-mVenus fusion in addition to endogenous cyclin D1-mVenus. Although this manipulation indeed shortened the average length of G1 phase from 7.7 ± 3.0 to 5.6 ± 1.5 hr (Figure S5E), cyclin D1-CDK4-mVenus levels did not correlate well with the length of G1 phase (Figure 6C), as observed for endogenous cyclin D1. We conclude that cyclin D1 is required for proliferation but is likely not the only time-limiting factor for the G1/S transition.

### Cyclin D1 Maintains G1 Phase and Prevents the Transition into Quiescence

Our observation that cyclin D1 levels are a poor predictor of G1 phase length supports a new model of G1 phase regulation in which cyclin D-CDK-dependent phosphorylation of Rb prevents cell cycle exit and differentiation (Narasimha et al., 2014). Indeed, depletion of cyclin D1 efficiently blocked proliferation (Figure 6A) and caused a loss of mRuby-PCNA within 48 hr, indicative of the transition into quiescence (Figure S5G). In agreement, cyclin-D1-depleted cells strongly accumulated the CDK2 activity sensor DHB-Venus in the nucleus (Figures 6D and S5F), indicating a CDK2^low^ state that has been proposed to represent G0 (Spencer et al., 2013). Further, chemical inhibition of CDK4 decreased mRuby-PCNA and cyclin A2-mVenus to an even greater extent than serum starvation. In agreement, we observed reduced Rb phosphorylation on serines 780 and 807/811 and fewer cells expressing the proliferation marker Ki67 (Gerdes et al., 1983; Figure 6E). On the contrary, arresting cells in G1 phase by nucleolin depletion (Kittler et al., 2007) blocked proliferation to a similar extent as cyclin D1 depletion but only mildly affected the levels of mRuby-PCNA (Figures 6A and S5G). Consequently, Rb phosphorylation and Ki67 staining were only reduced in cells with strongly affected mRuby-PCNA (Figure S6H). Taken together, these data highlight the utility of mRuby-PCNA as a quiescence marker for live-cell imaging and suggest that a key role of cyclin D-CDK4 is to maintain G1 phase and prevent cell cycle exit.

### Specific Cyclin D1 Kinetics Poises Cells for Short G1 Phases

Given that total population analysis revealed a poor correlation between cyclin D1 levels and G1 phase length, we wondered whether there was a common hallmark that distinguishes cells with short G1 phases from the rest. We observed that 14% of cells with continuously decreasing levels of cyclin D1 since mitosis (class I: Figures 5D and 6F) had significantly shorter G1 phases. Interestingly, these cells also displayed higher maternal cyclin D1 levels compared to the total population (Figure 6G), consistent with the possibility that the decision to proliferate or not took place at the maternal restriction point (Chen et al., 2013, Spencer et al., 2013). If this was the case, both sister cells ought to have similar cyclin D1 and G1 phase kinetics. However, 68% of sister cells developed distinct cyclin D1 kinetics after mitosis and consequently also differed in the length of G1 phase (Figure 6H). This was unlikely to be due to asymmetric segregation of cyclin D1 during mitosis because both sister cells started G1 phase with almost identical levels (rho = 0.92; Figure 6I). Hence, in most cells, even high levels of maternal cyclin D1 do not predict the length of G1 phase in the emerging daughter cells, thereby suggesting that other events contribute to the decision making. We conclude that there is a subpopulation of cells with distinct cyclin D1 kinetics that are potentially a hallmark of short G1 phases and note that this insight was made possible by the concurrent analysis of cell cycle and protein dynamics across two cell divisions.

## Discussion

Here, we present endogenously tagged PCNA as an all-in-one cell cycle reporter for advanced cell fate analyses in living cells. Overexpression of fluorescently tagged PCNA has been used before in cell culture and animal models (Leung et al., 2011) to assign G1, S, and G2 phases, but this method required a second marker, such as a fluorescently labeled histone, for automated segmentation (Piwko et al., 2010, Schmitz et al., 2010). Furthermore, continuous overexpression of such *PCNA* transgenes prevents identification of the crucial fate decision to exit from G1 phase into quiescence or differentiation, where only expression of endogenous *PCNA* ceases (Thacker et al., 2003, Yamaguchi et al., 1995). We show that endogenous mRuby-PCNA faithfully recapitulates the cell cycle expression and localization dynamics of the untagged allele, indicating that it is a bona fide marker of all cell cycle phases, including quiescence. Furthermore, deriving cell cycle kinetics from a single endogenous reporter rather than depending on the interplay of multiple overexpressed transgenes simplifies imaging and analysis workflows. In proliferating cells, PCNA-dependent segmentation, tracking, and fluorescence extraction is indistinguishable from that obtained with classical histone-based methods, thereby allowing simultaneous visualization of up to three additional proteins within the same cell without the need for advanced image-processing techniques.

Altogether, endogenously tagged PCNA as an all-in-one cell cycle reporter combines the best features of current fixed and live-cell analyses that cannot be achieved by either approach alone: (1) monitoring the behavior of multiple proteins in parallel in living cells in a cell-cycle-dependent and quantitative manner; (2) defining cause-consequence relationships by relating protein dynamics to cell cycle decisions; and (3) detecting the transition into quiescence and cell cycle re-entry in living cells.

Using increased PCNA abundance to determine the G1/S transition at high temporal resolution requires time-lapse experiments and robust single-cell tracking. This might be challenging in rapidly moving cells or more complex 3D structures and precludes precise cell cycle analysis from single snapshots. At least the latter issue can be circumvented by higher resolution microscopy to capture the first replication foci in S phase (Leonhardt et al., 2000). Because mRuby-PCNA expression ceases upon cell cycle exit, an additional segmentation marker is required for quiescence studies. Here, we have used an endogenous knockin of mTurquoise2 into the histone 3.1 locus to solve this issue, but standard ectopic expression of any fluorescently tagged DNA marker or a recently described non-toxic infrared DNA dye for live cell imaging (Lukinavičius et al., 2015) are easily accessible alternatives. Whereas creating an endogenous cell cycle reporter appears to be more laborious than traditional transgene-based reporters, we find that heterozygous PCNA reporters can be readily obtained in transformed, non-transformed, and primary cells in human and murine cell culture by rAAV-mediated homologous recombination within 1 month. With CRISPR/Cas9 technology coming of age and the advent of SPIM microscopy, we have no doubt that the presented methodology can be easily adapted to animal models and complex 3D cultures, such as organoids.

Focusing on three major cyclins and p21, we demonstrate how the endogenous all-in-one PCNA reporter can be used to correlate cell cycle and protein dynamics across multiple independent PCNA reporter lines and in response to perturbations. We provide a comparative and quantitative view of cyclin oscillations by endogenous tagging of all cyclins with the same fluorescent protein and standardized imaging procedures. Single-cell tracks from hundreds of individual cells confirm the well-characterized cell-cycle-dependent expression kinetics of cyclins A2 and B1 (Pines and Hunter, 1991) in the nucleus and cytoplasm, respectively, and place the reduction of nuclear cyclin D1 at the G1/S transition (Lukas et al., 1994, Pagano et al., 1994, Yang et al., 2006). Although the average cyclin oscillations and the response of p21 to DNA damage are in line with previous biochemical analyses and studies of fixed cells (Di Leonardo et al., 1994, el-Deiry et al., 1993, el-Deiry et al., 1994), the different endogenous PCNA reporters we introduce here as a resource present a unique opportunity to address questions of single-cell behavior that were previously out of reach. To illustrate the potential of this tool, we evaluated the role of cyclin D1 in G1 phase and restriction point regulation. This addresses two long-standing cell cycle paradigms that are currently challenged by new insights into the regulation of Rb activity (Narasimha et al., 2014) and the discovery of a maternal restriction point (Spencer et al., 2013).

First, we demonstrate that neither the expression levels of cyclin D1 nor of active cyclin D1-CDK4 correlate well with G1 phase length. This is at odds with the classical view that progressive hypophosphorylation of Rb (Ezhevsky et al., 2001, Mittnacht et al., 1994) by increasing amounts of active cyclin D-CDK4/6 complexes causes initial E2F activation and starts a cyclin-E-dependent positive feedback loop that drives cells into S phase (Johnson and Skotheim, 2013). On the other hand, formation of the active cyclin D-CDK complex appears to be one time-limiting step in G1 phase because constitutive expression of a cyclin D1-CDK4 fusion shortens the average duration of G1 phase. How can these findings be reconciled with the notion that increased cyclin D levels are a hallmark of many rapidly proliferating cancer cells (Alao, 2007)? One possible answer lies in our observation that cyclin D1 expression maintains G1 phase and prevents the transition into quiescence, presumably by keeping Rb in a monophosphorylated state (Narasimha et al., 2014). Hence, rather than accelerating the speed of the cell cycle directly, elevated levels of cyclin D might “prime” cells for cell cycle progression and thereby tilt the balance between proliferation and quiescence.

Second, we reveal distinct cyclin D1 kinetics in a subpopulation of cells that are characterized by short G1 phases. Though all of these cells are derived from parent cells with high levels of cyclin D1, suggestive of a decision at the proposed maternal restriction point (Chen et al., 2013, Spencer et al., 2013), we observe the emergence of heterogeneity in both cyclin D1 levels and G1 phase length despite the fact that both sister cells inherit the same amount of cyclin D1. Hence, whereas cells begin integrating proliferating signals prior to mitosis (Spencer et al., 2013, Stacey, 2003), determinants other than cyclin D1, e.g., asymmetric segregation of DNA damage, define G1 phase length (Barr et al., 2017).

In conclusion, our study demonstrates a means of precisely and simultaneously quantifying cell cycle kinetics and spatiotemporal behavior of multiple endogenous proteins at single-cell resolution. The different endogenous reporters and the image analysis pipeline described here provide a powerful resource to further address the role of cyclin D1 and p21 in the maternal restriction point and G1 phase regulation. Moreover, the all-in-one PCNA-based reporter will enable direct studies of cause-consequence relationships between cell cycle kinetics and cell fate determinants, e.g., during reprogramming and differentiation.

## Experimental Procedures

### Cell Culture and RNAi

All cells were cultured according to standard mammalian tissue culture protocols as described in more detail in the Supplemental Experimental Procedures. Site-specific integration of genes encoding fluorescent proteins into the genome was achieved by rAAV-mediated homologous recombination followed by flow cytometry sorting of single cells as described previously (Collin et al., 2013). Cells were transfected with endoribonuclease-prepared siRNA (esi-RNA) oligos (EUPHERIA Biotech) targeting cyclin D1 (EHU153321) or nucleolin (EHU080431) at 0.35 ng/μL using Lipofectamine RNAiMAX transfection reagent (Thermo Fisher Scientific) according to the manufacturer’s instructions and analyzed after 48 hr by live-cell imaging or immunofluorescence.

### Quantitative Western Blotting

Relative levels of tagged and untagged cyclins and cyclins bound to CDKs were determined by western blotting using an Odyssey quantitative infrared scanning system (LI-COR Biosciences) as described in detail in the Supplemental Experimental Procedures.

### Induction of Cell Cycle Exit and Quiescence and Drug Treatment

For serum starvation, asynchronously growing cells were washed twice with PBS followed by addition of the respective cell culture medium containing the indicated amount of FBS for 48 hr: hTERT RPE-1 and FL83B (0% FBS); MCF10A and HeLa (0.3% FBS); and BJ fibroblasts (0.3% FBS). For contact inhibition, cells were grown to confluence within 5–8 days. DNA damage was induced by the addition of 1 μM etoposide (Sigma-Aldrich; E1383) followed by live-cell analysis for 24.5 hr, and CDK4 was inhibited by 0.5 μM PD0332991 (Sigma-Aldrich; PZ0199).

### Semiquantitative PCR Analysis

Total RNA was isolated from asynchronous and serum-starved RPE-1 cells using the High Pure RNA Isolation kit (Roche) according to the manufacturer’s protocol and then cDNA was synthesized using Affinity Script (Agilent Technologies) and analyzed according to the 2^−ΔΔCt^ method (Livak and Schmittgen, 2001) as described in detail in the Supplemental Experimental Procedures.

### Immunofluorescence

RPE-1 cells were plated on glass coverslips 24 hr before serum withdrawal. After 48 hr serum starvation, cells were fixed using 4% paraformaldehyde in PBS and permeabilized with CSK buffer (25 mM HEPES [pH 7.8], 50 mM NaCl, 1 mM EDTA, 3 mM MgCl_2_, 300 mM sucrose, and 0.2% Triton X-100) for 10 min. Phosphorylated Rb was detected with anti-pRb Ser780 (D59B7; rabbit monoclonal antibody [mAb] 8180; Cell Signaling Technology; 1:1,000), Ser807/811 (D20B12; rabbit mAb 8516; Cell Signal; 1:2,000), and anti-Ki67 (rabbit mAb MA1-9084; Pierce; 1:2,000) antibodies.

### Imaging

Automated time-lapse microscopy was performed using an ImageXpress Micro XLS wide-field screening microscope (Molecular Devices) equipped with 10×, 0.5 numerical aperture (NA); 20×, 0.7 NA; and 40×, 0.95 NA Plan Apo air objectives (Nikon) and a laser-based autofocus as described in detail in the Supplemental Experimental Procedures.

### Automated Image Analysis

Single-cell segmentation and classification was performed with Mathematica 10.4 (Wolfram Research). Custom Fiji plugins (Schindelin et al., 2012) and CellTracker software (Scherf et al., 2013) were used for tracking. Data analysis and visualization were done with Mathematica 10.4 (Wolfram Research). All components of our pipeline are described in detail in the Supplemental Experimental Procedures, and the corresponding scripts are available upon request.

### Statistical Methods

Statistical analysis was done with Mathematica 10.4 (Wolfram Research) using Mann-Whitney U test for pairwise comparisons and Spearman’s rho for correlation analyses.

## Author Contributions

Conceptualization, J.M.; Methodology, T.Z., I.A.G., D.K., K.D., D.M., J.M., and I.G.; Software, T.Z.; Investigation, T.Z., I.A.G., D.K., K.D., M.G., M.F., and J.M.; Writing – Original & Revised Draft, J.M.; Writing – Review & Editing, T.Z., I.A.G., J.M., and I.G.; Funding Acquisition, J.M. and I.G.; Supervision, J.M. and I.G.

## Supplemental Information

Document S1. Supplemental Experimental Procedures and Figures S1–S6

Movie S1. Single-Cell Tracking Based on PCNA, Related to Figure 4 Exemplary single cell tracks derived from segmentation and tracking using mRuby-PCNA.

Movie S2. Cyclin A2-mVenus Dynamics during a Complete Cell Cycle, Related to Figures 3 and 5 Representative quantification of mRuby-PCNA and Cyclin A2-mVenus dynamics from mitosis to mitosis.

Movie S3. Cyclin B1-mVenus Dynamics during a Complete Cell Cycle, Related to Figure 5 Representative quantification of mRuby-PCNA and Cyclin B1-mVenus dynamics from mitosis to mitosis.

Movie S4. Cyclin D1-mVenus Dynamics during a Complete Cell Cycle, Related to Figure 5 Representative quantification of mRuby-PCNA and Cyclin D1-mVenus dynamics from mitosis to mitosis.

Movie S5. mAG-hGem and mRuby-PCNA Dynamics during a Complete Cell Cycle, Related to Figure S2 Representative quantification of mRuby-PCNA and mAG-hGem (FUCCI) dynamics from mitosis to mitosis.

Document S2. Article plus Supplemental Information
