# Supplementary material for: Quantitative Cell Cycle Analysis Based on an Endogenous All-in-One Reporter for Cell Tracking and Classification
Source: Cell Rep. 2017 May 30;19(9):1953–66. doi: 10.1016/j.celrep.2017.05.022 (PMC5464964; doi:10.1016/j.celrep.2017.05.022)
Supplement: Document S1. Supplemental Experimental Procedures and Figures S1–S6 [file mmc1.pdf]

Cell Reports, Volume *19*

Supplemental Information

Quantitative Cell Cycle Analysis Based on an Endogenous All-in-One Reporter for Cell Tracking and Classification

Thomas Zerjatke, Igor A. Gak, Dilyana Kirova, Markus Fuhrmann, Katrin Daniel, Magdalena Gonciarz, Doris Müller, Ingmar Glauche, and Jörg Mansfeld

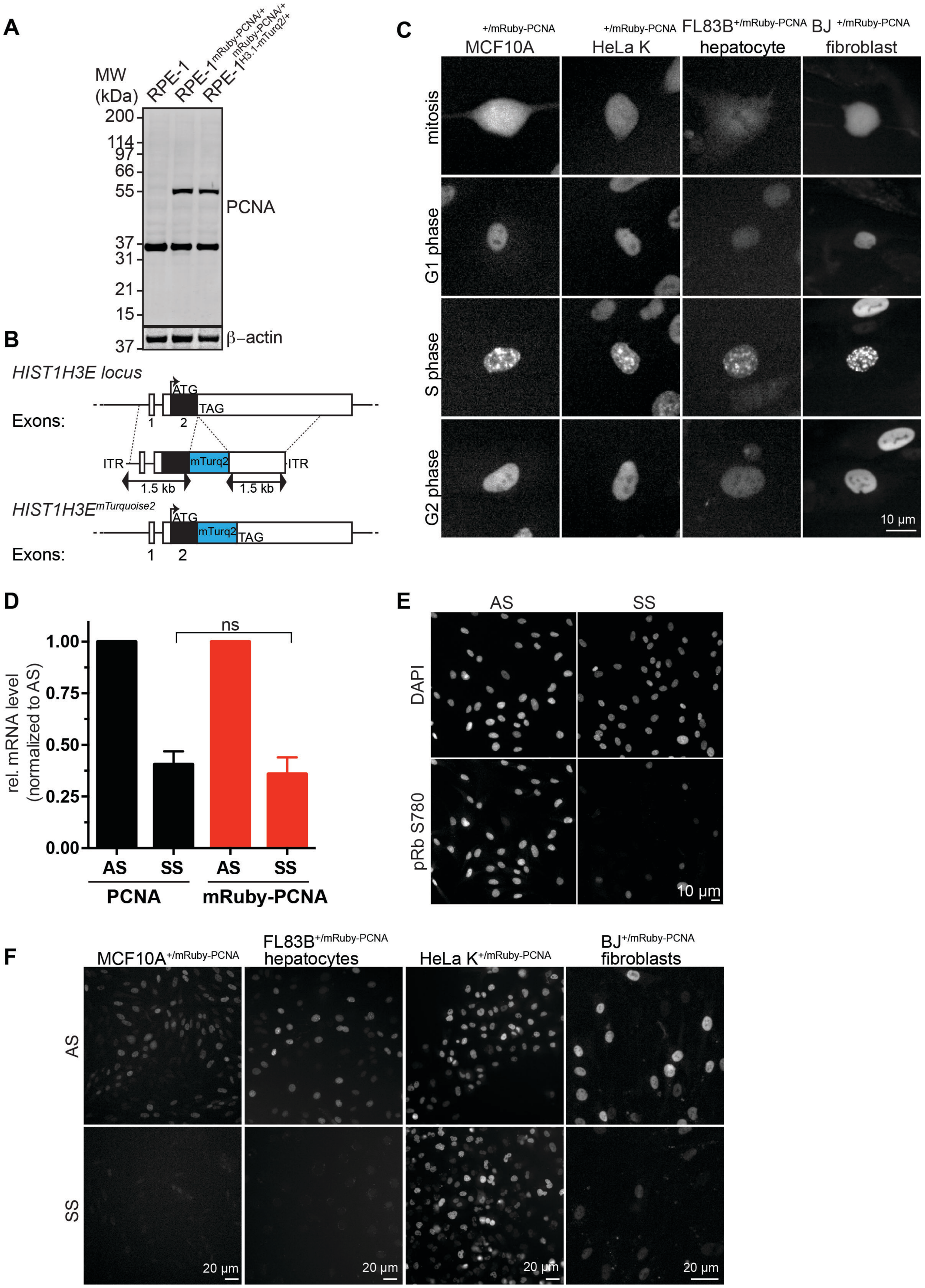

Figure S1, related to Figures 1 & 2. Histone 3.1 targeting and mRuby-PCNA reporters in non-transformed and transformed cells. (A) Representative Western analysis with the indicated antibodies confirming heterozygous targeting of PCNA. (B) C-terminal targeting of endogenous Histone 3.1 with mTurquoise2. (C) Cell cycle-dependent localization of endogenous mRuby-PCNA in non-transformed human MCF10A and BJ foreskin fibroblasts, murine FL83B hepatocytes and transformed HeLa K cells. (D) qPCR analysis of asynchronous (AS) and 48-hour serum starved RPE-1 cells (SS) showing a similar reduction of PCNA and mRuby-PCNA transcripts. Data are represented as mean ± SEM from three independent experiments (E) Immunocytochemistry for phospho Rb S780 in RPE-1 cells treated as in (D). (F) mRuby-PCNA expression in response to serum starvation in the indicated living cell lines treated as in (D).

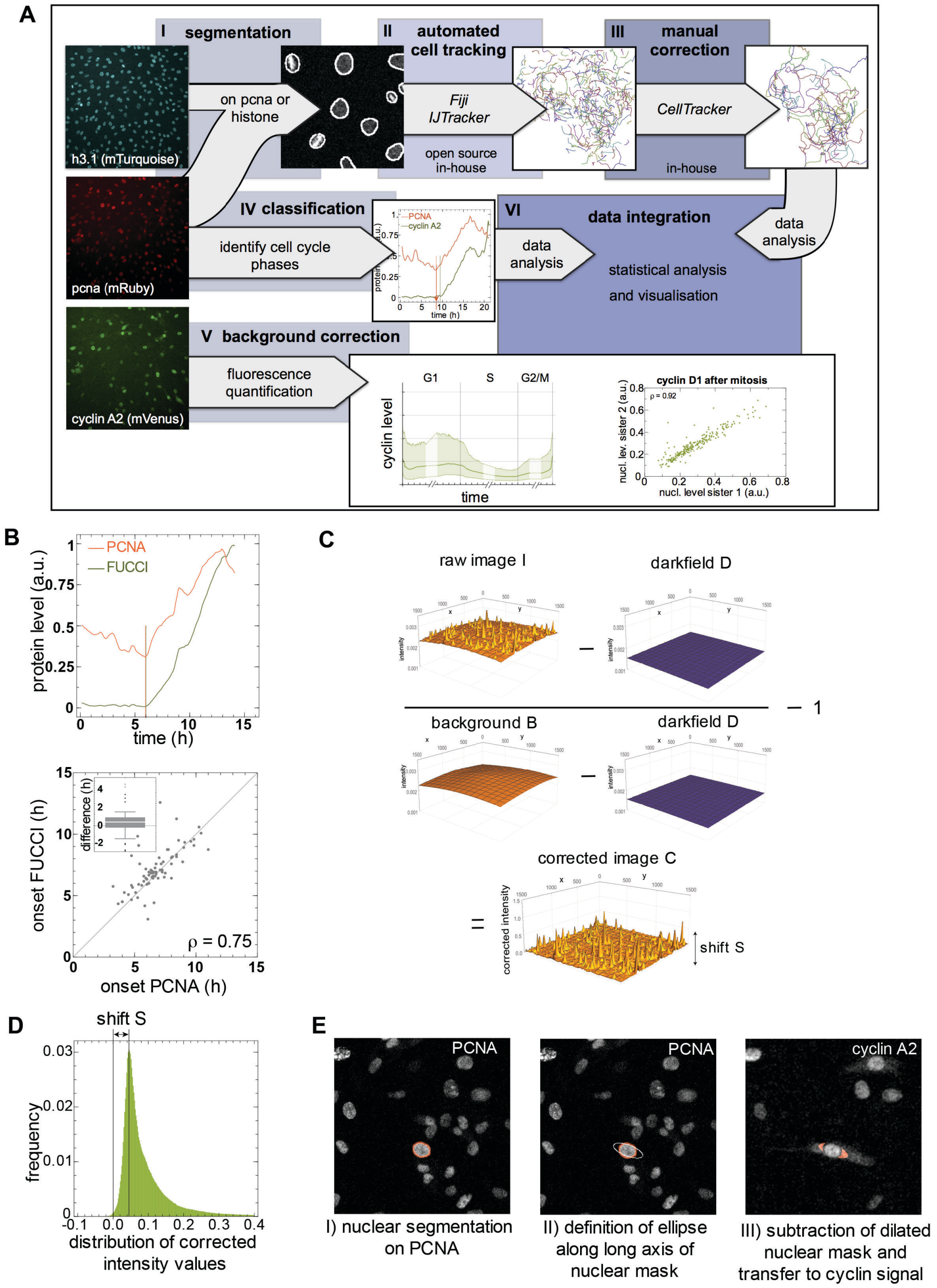

Figure S2, related to Figures 3 & 4. Quantitative image analysis pipeline. (A) Sketch of workflow for segmentation (I) and tracking (II-III) of single cells, estimation of cell cycle phases (IV) and quantification of fluorescent protein levels (V). **(B)** Representative single cell track and scatter plot illustrating the correlated onset of mRuby-PCNA and FUCCI (mAG-hGem) expression. Note that mRuby-PCNA precedes FUCCI (mAG-hGem) on average by 16 minutes (insert) and is used to define the beginning of S phase (n=73). See also Supplemental Movie 5. **(C)** Background correction method for quantitative read-out of fluorescence intensities. Subtracting the darkfield image *D* accounts for the camera-specific offset (i.e. the returned signal without any source of light). To account for uneven illumination, images are divided by background images that were derived by averaging over several time series of empty wells (containing only medium). **(D)** To account for differences between background images *B* and the individual background of the images *I*, a well-specific shift *S* is calculated and subtracted from the corrected images *C*. Therefore, the mode of the intensity distribution of each image *C* (i.e. the peak of the distribution) is assumed to be the mean background and is shifted to 0. **(E)** Quantification of cytoplasmic fluorescence. As major parts of the cytoplasm are often located at the poles of the nucleus (I), an ellipse is estimated that is prolonged along the larger nuclear axis and shortened perpendicular to it (II). The nuclear mask is expanded and subtracted from the ellipse, leaving two regions at the poles that are used to quantify cytoplasmic fluorescence (III).

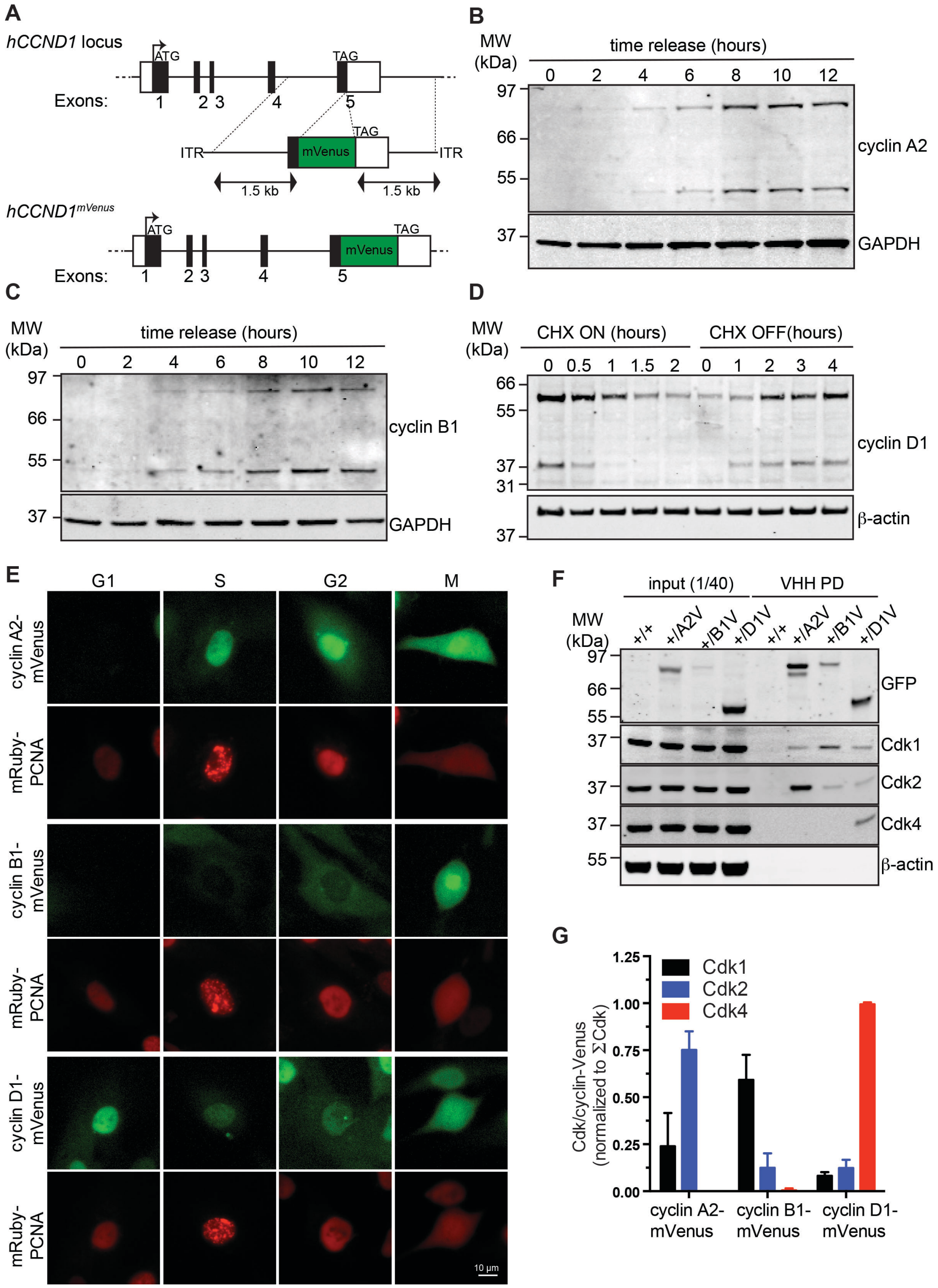

Figure S3, related to Figure 5. Cyclin D1 targeting and characterization of cyclin-mVenus cell lines. (A) C-terminal targeting of endogenous cyclin D1 with mVenus. **(B, C)** Representative Western analysis with the indicated antibodies of a release from a G1/S thymidine arrest showing the behavior of tagged and untagged cyclins A2 and B1. **(D)** Representative Western analysis with the indicated antibodies to monitor the levels of tagged and untagged cyclin D1 during a cycloheximide (CHX) block and release. **(E)** Snapshots from living cells illustrating the cell cycle-dependent expression of cyclins B1, and D1-mVenus in the background of the endogenous PCNA reporter. **(F)** mVenus-tagged cyclins A2, B1 and D1 from asynchronously growing RPE-1 cells were precipitated with GFP-binder beads, separated by SDS-PAGE and analyzed for binding of CDK1, CDK2 and CDK4 by quantitative Western blotting using the indicated antibodies. **(G)** Quantification of three independent experiments as shown in (F). CDK binding normalized to the amount of precipitated mVenus-tagged cyclin is expressed as the sum of the total signal for the respective CDK. Data are represented as mean ± SEM.

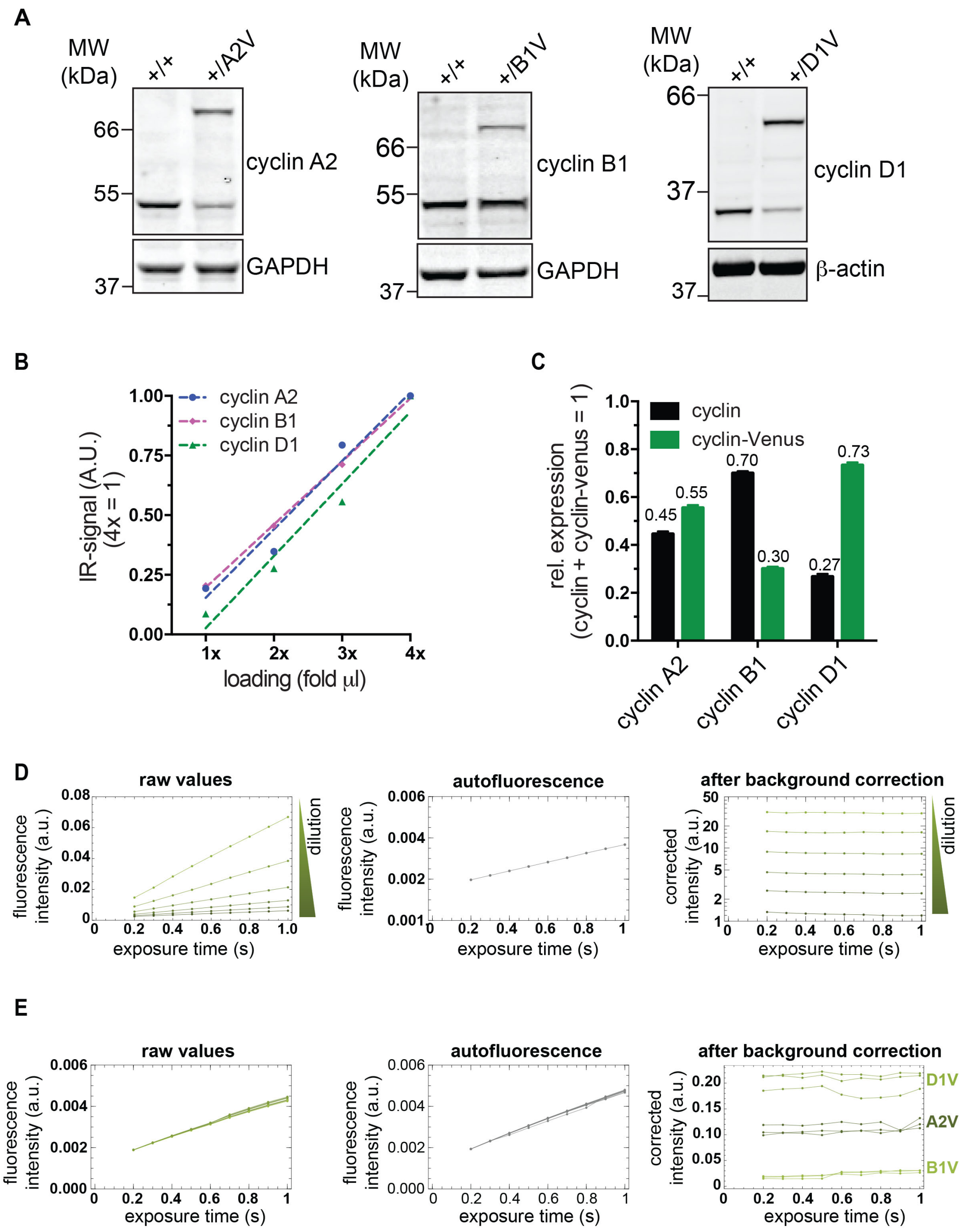

Figure S4, related to Figure 5. Quantification of tagged and untagged cyclin levels and quantitative imaging. **(A)** Quantitative Western analysis with the indicated antibodies comparing the relative expression levels of tagged and untagged cyclins in asynchronously growing RPE-1 cells. **(B)** Quantification of the linear relationship between the amount of extract loaded and the recorded near-infrared intensity derived from Western analyses using the same antibodies as in (A). **(C)** Quantification of the relative expression level of tagged and untagged cyclins from the experiments shown in (A). The observed differences between the two alleles were used to derive the total amount of cyclins for the single cell analyses shown in Figures 5A-C. Data are represented as mean ± SEM from three independent experiments with at least three technical repeats. **(D)** Illumination time-dependent fluorescence intensity of different concentrations of recombinant mVenus diluted in imaging medium compared to the autofluorescence of imaging medium alone. **(E)** Illumination time-dependent fluorescence intensity of mVenus-tagged cyclins compared to the autofluorescence of imaging medium alone. Note, because mVenus and autofluorescence scale in the same manner, the recorded signal after background correction (see Supplemental Experimental Procedures) becomes independent of the exposure time for both recombinant mVenus diluted in imaging medium (D) and cyclin-mVenus expressed in cells (E). This enables comparison of relative cyclin expression levels even when different exposure times are used.

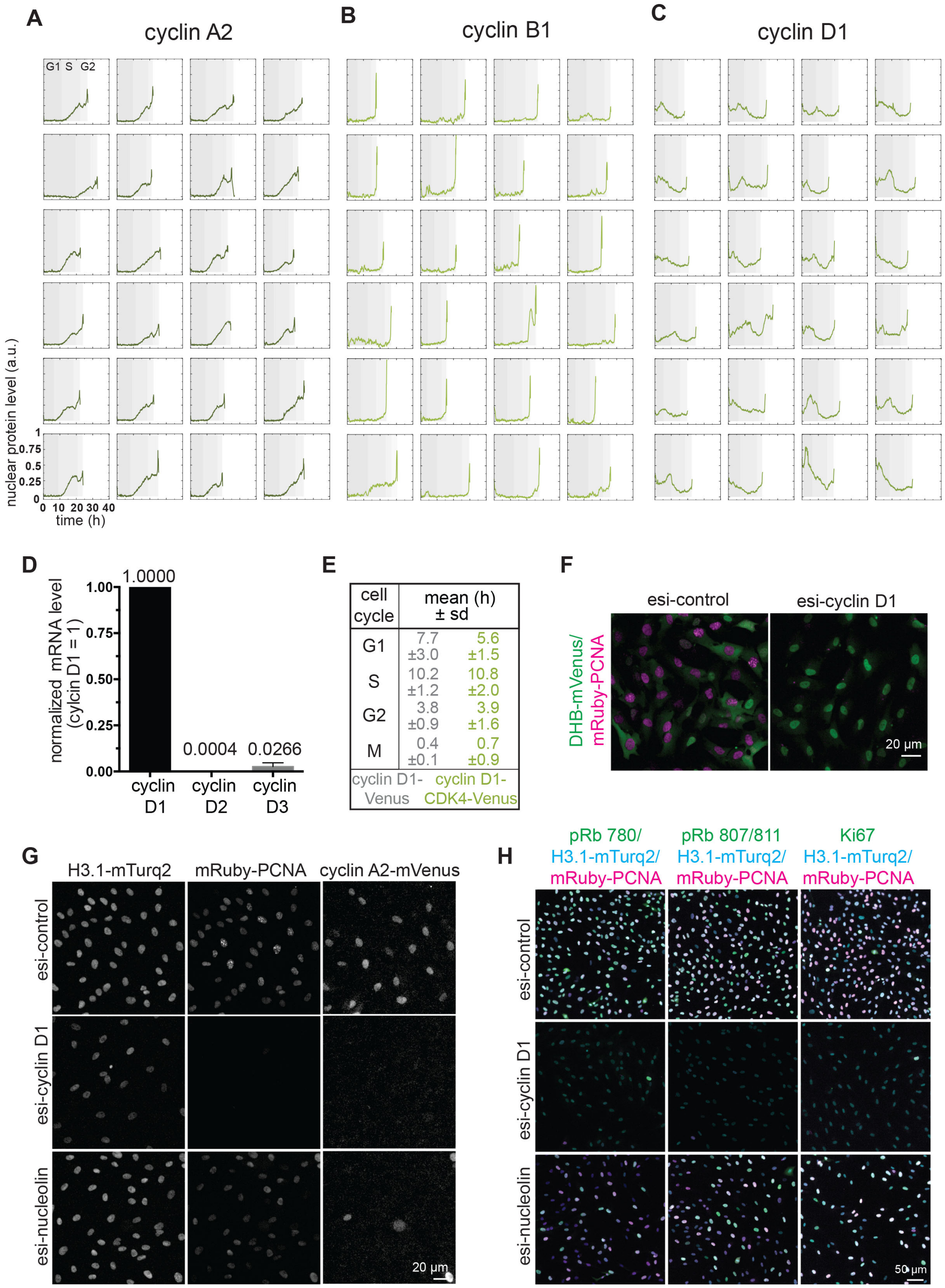

Figure S5, related to Figures 5 & 6. Oscillations of cyclins in single cells and cell cycle dynamics of CDK4-cyclin D1-mVenus expressing cells. (A-C) Representative single-cell tracks from five-minute time-lapse experiments showing quantitative cyclin oscillations from mitosis to mitosis. **(D)** qPCR analysis showing that cyclin D1 is the predominant D-type cyclin expressed in RPE-1(hTERT) cells. Data represent mean values of three independent experiments normalized to cyclin D1. Error bars indicate SD. **(E)** Table showing cell cycle phase timing of RPE-1 cells expressing endogenous cyclin D1-mVenus or endogenous cyclin D1-mVenus and a constitutively expressed ectopic cyclin D1-CDK4-mVenus fusion, respectively. **(F)** Representative images of living cells expressing the CDK2 sensor DHB-mVenus treated with the indicated esi-RNA oligos for 48 hours. Note, cyclin D1-depleted cells have strongly reduced levels of mRuby-PCNA and accumulate DHB-mVenus in the nucleus indicative of a quiescent CDK2^{low} state. **(G)** Representative images of living cells treated with the indicated esi-RNA oligos for 48 hours showing that depletion of cyclin D1 but not nucleolin strongly reduces mRuby-PCNA. **(H)** Immunostaining of cells treated as in (G) with pRB and Ki67 antibodies.

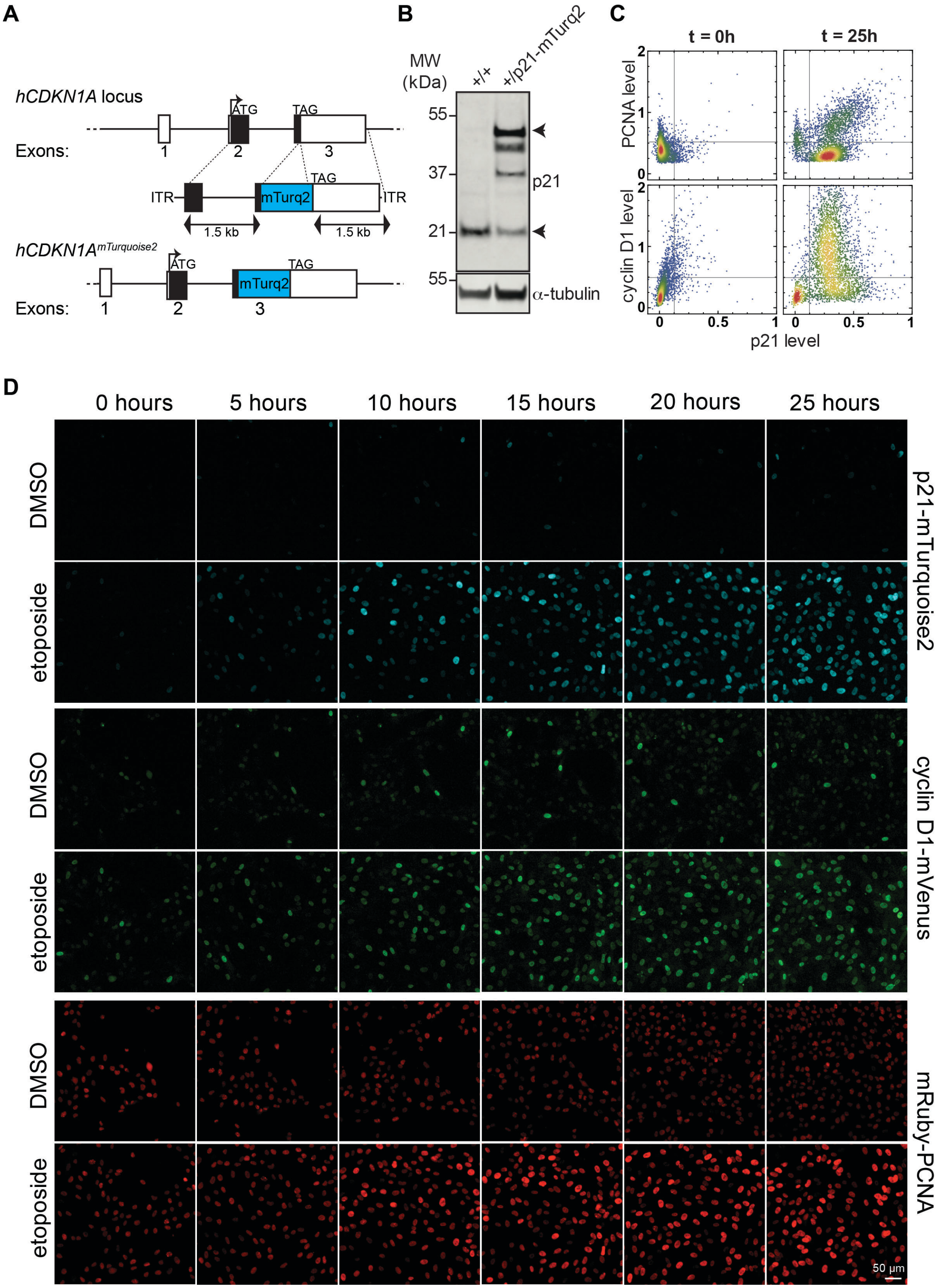

Figure S6, related to Figure 5. Targeting of p21 and response of p21, cyclin D1 and PCNA to DNA damage. **(A)** C-terminal targeting of endogenous p21 with mTurquoise2. **(B) R**epresentative Western analysis using the indicated antibodies of endogenous mRuby-PCNA/cyclin D1-mVenus/histone3.1-mTurquoise2 and mRuby-PCNA/cyclin D1-mVenus/p21-mTurquoise2 reporters showing heterozygous targeting of p21 with mTurquoise2. **(C)** Quantification of p21-mTurquoise2, cyclin D1-mVenus and mRuby-PCNA levels in living cells treated with 1 µM etoposide for the indicated time. **(D)** Montage of time-lapse images of cells treated as in (C).

Supplemental Experimental Procedures

Cell Culture

Parent hTERT RPE-1 cells, hTERT RPE-1 cyclin A2-mVenus and hTERT RPE-1 cyclin B1-mVenus were a kind gift from Jonathon Pines (ICR, London, UK), parent MCF10a cells from Chris Bakal (ICR, London, UK), and FL83B hepatocytes from Marc Bickle (MPI-CBG Dresden, Germany). BJ-fibroblasts were obtained from the American Type Culture Collection (ATCC). hTERT RPE-1 cells were cultured in DMEM/F12 (Sigmaaldrich, St. Louis, MO, USA) supplemented with 10% (v/v) FBS, 1% (v/v) penicillin-streptomycin, 1% (v/v) Glutamax, 0.5 μg/mL Amphotericin B and sodium bicarbonate. HeLa cells were maintained in Advanced DMEM (Gibco) supplemented with 2% FBS, 1% (v/v) penicillin-streptomycin, 1% (v/v) Glutamax and 0.5 μg/mL Amphotericin B. MCF10A cells were cultured in DMEM/F12 (Invitrogen, Carlsbad, CA) supplemented with 5% FBS, 1% (v/v) penicillin-streptomycin, 20 ng/mL EGF, 0.5 mg/mL hydrocortisone, 10μg/mL insulin and 100 ng/mL cholera toxin. BJ fibroblasts were grown in Eagle's Minimum Essential Medium (EMEM) (LGC Standards, Teddington, UK)) supplemented with 10% FBS and 1% (v/v) penicillin-streptomycin. FL83B were cultured in F-12K Nut Mix (Gibco) supplemented with 10% FBS and 1% (v/v) penicillin-streptomycin. To generate stable cell lines expressing active CDK4-cyclinD1 complexes, the endogenous RPE-1 mRuby-PCNA/cyclin D1-mVenus/histone 3.1-mTurquoise reporter was electroporated with a plasmid encoding a fusion of Flag-tagged human CDK4, murine cyclin D1 and mVenus tagged with a hemagglutinin (HA) tag and a minimized auxin-inducible degron, followed by selection for stable integrants with 400 μg/ml neomycin.

Quantitative Western blotting and GFP-binder pull down

For determination of the relative amount of tagged and untagged cyclins, cells and GFP-Binder pull downs were directly lysed in 1x NuPAGE LDS buffer (Thermo Fisher Scientific, Waltham, MA, USA) or extracted on ice for 25 minutes in 30 mM Hepes pH=8.0, 175 mM NaCl, 2.5 mM $MgCl_2$, 0.5 % NP-40 supplemented with 1 mM PMSF, 1mM DTT and an EDTA-free Protease inhibitor cocktail (Sigmaaldrich), followed by clearance of lysates at 16,000 g for 15 minutes. Note that the signal for cyclin detection using the indicated antibodies showed a linear relationship with the extract loaded in the range used for comparison of tagged and untagged cyclins (Figure S4B). mVenus-tagged cyclins were precipitated with a recombinantly expressed GFP-binder (kind gift of Thomas Surrey, Francis Crick Institute London, UK) cross-linked to Dynabeads M-270 carboxylic acid (Thermo Fisher Scientific), washed extensively with 50 mM tris-HCl pH=8.0, 250 mM NaCl, 0.5% NP-40 and eluted with 1x NuPAGE LDS buffer. Proteins were separated by SDS-PAGE and analyzed by Western blotting using quantitative infrared scanning system (Odyssey, LICOR, Lincoln, Nebraska, USA) with the indicated antibodies: anti-β-actin (Sigmaaldrich, A5441, 1/5000), anti-GAPDH (Santa Cruz, Dallas, Texas, USA. sc-20357, 1:1000), anti-PCNA (Cell Signaling, Danvers, MA. 13110, 1:1000), anti-cyclin B1 (GNS1, BD Pharmingen, San Jose, CA, USA. 554177, 1:1000), anti-cyclin A2 (custom mouse monoclonal, 1:1000), anti-cyclin D1 (DCS) (Santa Cruz, sc-20044, 1:200), anti-α-tubulin (Sigmaaldrich, T5167, 1:5000), anti-CDK1 (BD Biosciences, 610038, 1:5000), anti-CDK2 (BD Biosciences, 610146, 1:2500), anti-CDK4 (Cell Signaling, 12790, 1:1000), anti-p21 (Cell Signaling, 2947, 1:1000), anti-GFP (custom MPI-CBG, 1:5000). Note that the signal for cyclin detection using the indicated antibodies showed a linear relationship with the extract loaded in the range used for comparison of tagged and untagged cyclins (Figure S4B).

Semi-quantitative qPCR analysis

Relative expressions of the endogenous PCNA gene and the mRuby tagged allele were quantified using SYBR®Green-based qPCR kit (Promega, Madison, WI, USA) using the following primers: GAPDH forward 5'-AGATCCCTCCAAAATCAAGTGG-3', GAPDH reverse 5'-GGCAGAGATGATGACCCTTTT-3', PCNA forward 5'-CTAAAATGCGCCGGCAATGA-3', PCNA reverse 5'-TCTCCTGGTTTGGTGCTTCA-3', mRuby-PCNA forward 5'-AAAGGCTGGAAGAGTCCG-3', mRuby-PCNA reverse 5'-GGACATACTGGTGAGGTTCACG-3', cyclin D1 forward 5'-GACCCCGCACGATTTCATTG-3', cyclin D1 reverse 5'-AATGAACTTCACATCTGTGGCA-3', cyclin D2 forward 5'-GAGGCGGTGCTCCTCAATAG-3', cyclin D2 reverse 5'-CTCACAGGTCGATATCCCGC-3', cyclin D3 forward 5'-TGGAGACTGGCTCTGTTCGG-3', cyclin D3 forward 5'-GCTCCTCACATACCTCCTCGT-3'.

Imaging

Automated time-lapse microscopy was performed using an ImageXpress Micro XLS wide-field screening microscope (Molecular Devices, Sunnyvale CA, USA) equipped with a 10x, 0.5 N.A., 20x, 0.7 NA, and 40x, 0.95 NA Plan Apo air objectives (Nikon, Tokyo, Japan) and a laser - based autofocus. During the experiment, cells were maintained in a stage incubator at 37°C in a humidified atmosphere of 5% CO_2. All cell lines were grown in 96-well plastic bottom plates (μclear, Greiner Bio-One, Kremsmünster, Austria). Live-cell imaging was performed in the specific medium for the cell line, except for RPE-1 cells, where a modified DMEM containing 10% (v/v) FBS, 1% (v/v) penicillin-streptomycin, 1% (v/v) Glutamax, and 0.5 μg/mL Amphotericin B was used without phenol red and riboflavin to reduce auto fluorescence (Schmitz and Gerlich 2009). Images of the cells were acquired every 5 minutes for time courses of 48 h using a Spectra X light engine

(Lumencor, Beaverton, OR, USA), and a sCMOS (Andor, Concord, MA, USA) camera with binning = 1 and the indicated filter setup: CFP (Ex: 438/24; Dic: 426-450/467-600; Em: 483/32), TXRed (Ex: 562/40; Dic: 530-585 / 601-800; Em: 624/40), and YFP (Ex: 500/24; Dic: 488-512 / 528-625; Em: 542/27). Exposure times (cyclin A2-mVenus and cyclin D1-mVenus, 300 ms; cyclin B1-mVenus, 500 ms) for an optimal signal-to-noise ratio during quantitative mVenus imaging were determined according to a dilution series of recombinant mVenus and different exposure times of cyclin-mVenus-expressing cells as shown in Figures S4D and S4E.

Segmentation and tracking

Cell nuclei were segmented based on histone or PCNA expression. To estimate background fluorescence, we imaged several wells that only contained medium. Those sequences were used to compute a time course of background intensities. To correct for uneven illumination, we used the ratio between raw images and background for further analysis. Cell nuclei were segmented by thresholding with a subsequent watershed filtering. Single cell tracking was performed using a nearest neighbor method implemented in Fiji (Schindelin et al. 2012). Subsequently, we included a manual post-processing step to eliminate tracking errors and obtain reliable tracks of complete cell cycles. For this, we used an enhanced version of the software tool CellTracker (Scherf et al. 2013).

In contrast to the histone signal, PCNA expression changes considerably during cell cycle. Using this channel for both tracking and shape segmentation of nuclear boundaries requires an additional re-segmentation step. Whereas the global watershed segmentation is sufficient for localization of the cell object, we applied a secondary local adaptive binarization in a window of 100x100 pixels that was defined around the cell centroid. Mean and standard deviation of intensity values allowed estimation of an individual threshold for each cell object. This step improves segmentation both for low PCNA intensities during G1 phase and for high intensities during G2 phase.

A partial segmentation of the cytoplasm was obtained by fitting an ellipse along the longer axis of the segmented nucleus and subtracting the dilated nuclear mask (Fig. S2E).

Quantification of intensity levels

For background correction, the camera offset D (obtained as a darkfield image) was subtracted from both the time series of fluorescence images I_t and the time series of background images B_t (obtained by imaging wells that contain no cells but medium). The corrected images C_t were then defined as the ratio of pixel intensities between raw image and background image, subtracted by 1:

$$C_t = \frac{I_t - D}{B_t - D} - 1$$

Intensity values of 0 correspond to background intensity, whereas values of 1 correspond to intensities that are twice as high (Fig. S2C).

Differences in mean intensities between different image sequences with or without cells may result from different pH levels or fluorescence secretion by cells themselves. These moderate differences between the median background and the actual images were accounted for by estimating an individual shift S_t for each well and each time point t, which was additionally subtracted from the intensity values:

$$C_t^S = \frac{I_t - D}{B_t - D} - 1 - S_t$$

We obtained S_t as the mode of the distribution of intensity values (i.e. the highest peak), as we suppose this to be the mean background level (Fig. S2D). The corrected images C_t^S were then used to read out the cyclin kinetics of each tracked cell by taking the mean fluorescent signal within each segmentation mask, i.e. by dividing the sum of all pixel intensities by the cell area (see supplemental movies).

Classification of cell cycle phases
For all cells that were tracked completely from mitosis to mitosis, cell cycle phases were classified according to the following criteria, which are solely based on the time courses of PCNA expression and can be detected in an automated manner.

1. *Transition from G1 to S phase* (cf. Fig. 3B and 3C): Mean PCNA expression decreases during G1 and starts to increase at transition to S phase. We detect this reversal by a local linear regression (broken-stick approach). In particular, for each time point we determined two linear regressions using all measurements two hours prior to and two hours after this point. The transition between G1 and S phase was assigned to the point with the smallest angle between the two regressions, in which the second slope increases. This corresponds to the onset of increasing PCNA expression.

2. *Transition from S to G2 phase* (cf. Fig 3D and 3E): PCNA replication foci are formed during S phase and disappear at the transition from S to G2. These changes are reflected in the heterogeneity of PCNA pixel intensities, which can be quantified with the range between the 1% and 99% quantiles of the pixel intensity distribution. The time point of transition from S to G2 is defined as the point when the intensity range decreased from its highest peak.

3. *Transition from G2 to mitosis* (cf. Fig. 3F and 3G): At the start of mitosis, the nucleus dissolves and PCNA is distributed into the cytoplasm. This is reflected in a sharp decrease of the mean intensity level. The time point of transition from G2 to mitosis is defined as the start of the decrease.

After classification, all time courses were manually checked for consistency. Those that showed ambiguous signals or clear misclassification were excluded from further analysis.

Software
Single cell segmentation was done with Mathematica 10.4 (Wolfram Research Inc., Long Hanborough, UK). A self-written plug-in in Fiji (Schindelin et al. 2012) and in-house software CellTracker (Scherf et al. 2013) were used for tracking. Data analysis and visualization were done with Mathematica 10.4 (Wolfram Research Inc.), Prism (GraphPad, La Jolla, CA, USA) and ImageStudioLite (LI-COR).
