# Supplementary figures and images for: Quantitative Cell Cycle Analysis Based on an Endogenous All-in-One Reporter for Cell Tracking and Classification
Source: Cell Rep. 2017 May 30;19(9):1953–66. doi: 10.1016/j.celrep.2017.05.022 (PMC5464964; doi:10.1016/j.celrep.2017.05.022)

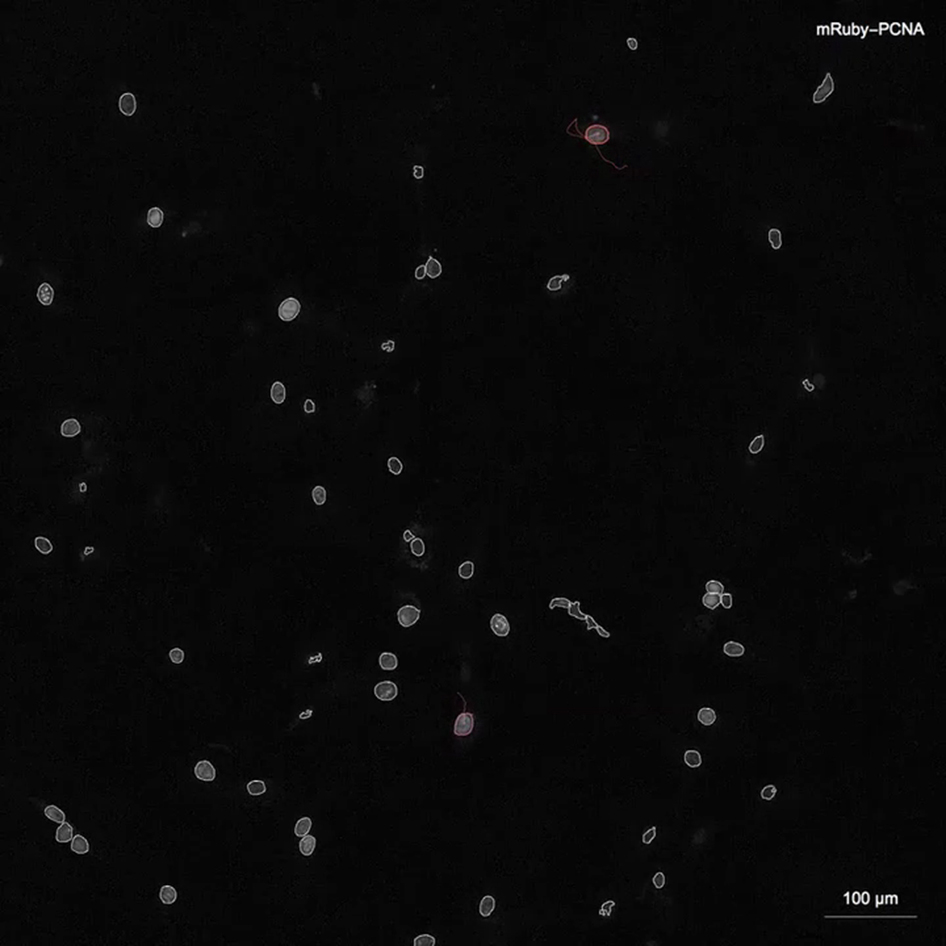

Supplement: Movie S1. Single-Cell Tracking Based on PCNA, Related to Figure 4 — Exemplary single cell tracks derived from segmentation and tracking using mRuby-PCNA. [file mmc2.jpg]

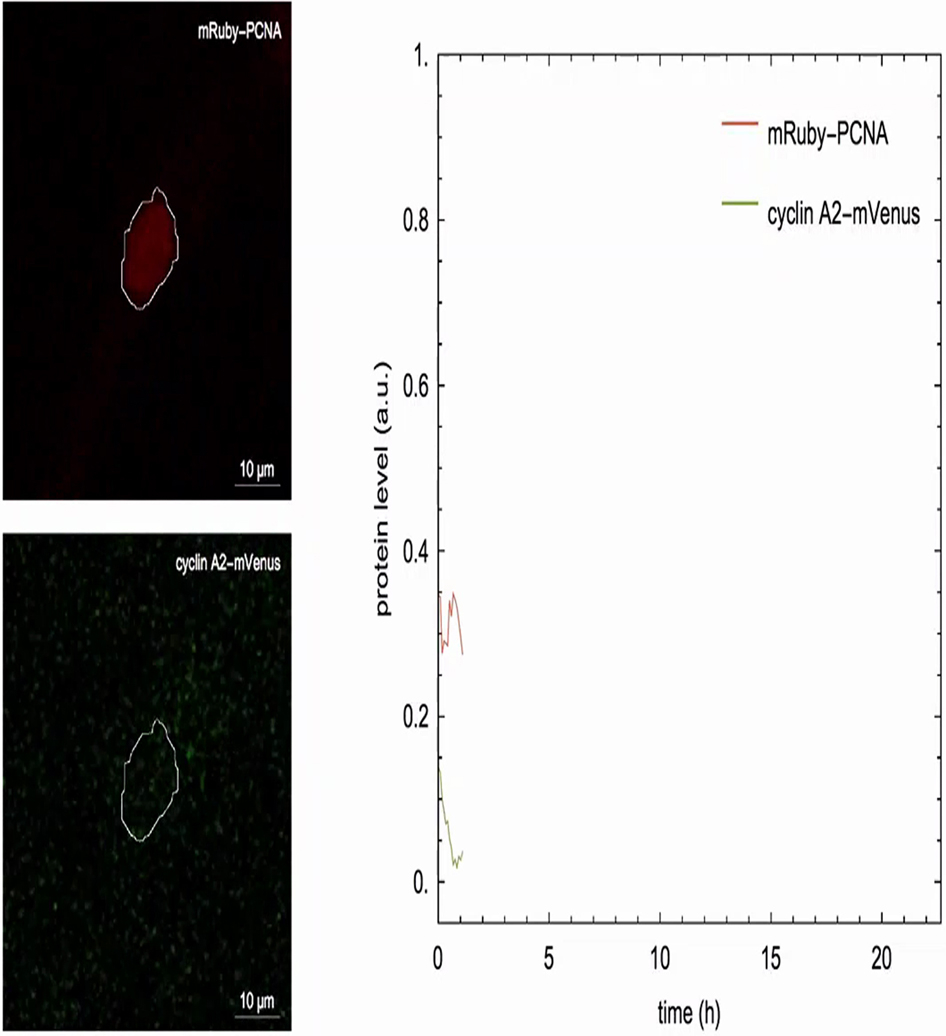

Supplement: Movie S2. Cyclin A2-mVenus Dynamics during a Complete Cell Cycle, Related to Figures 3 and 5 — Representative quantification of mRuby-PCNA and Cyclin A2-mVenus dynamics from mitosis to mitosis. [file mmc3.jpg]

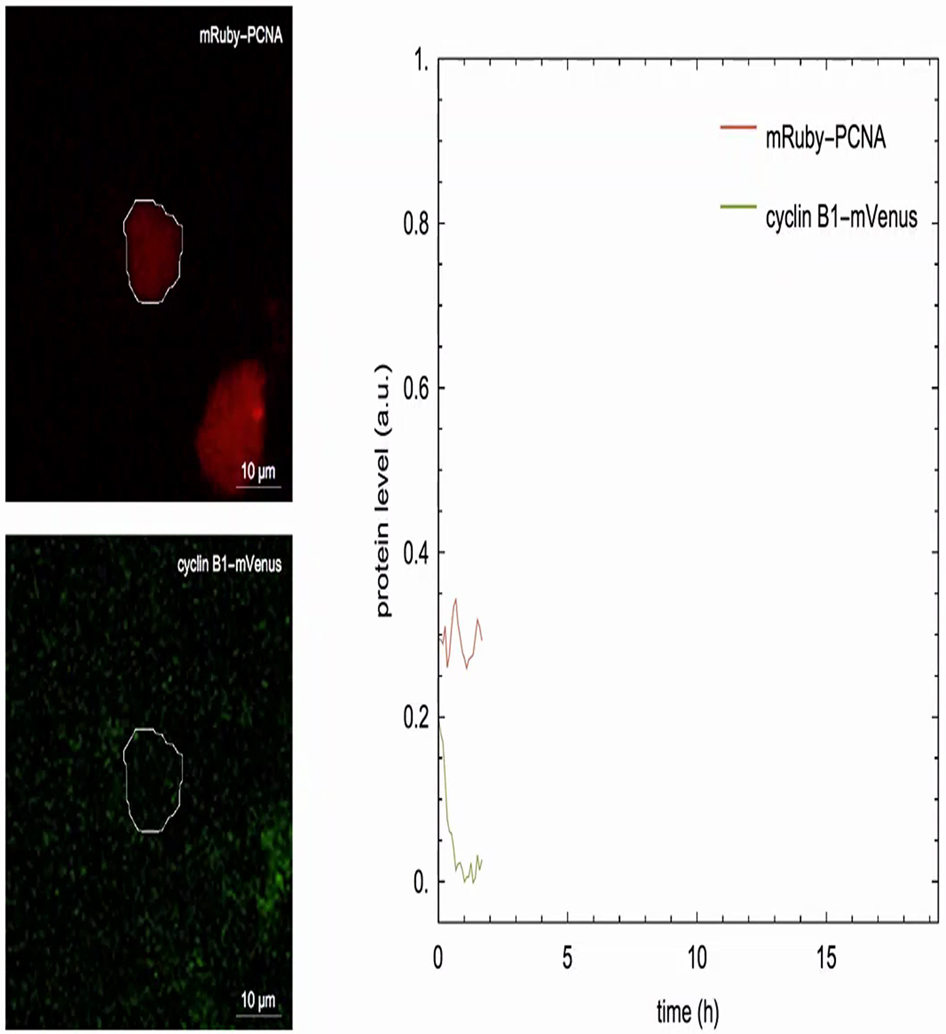

Supplement: Movie S3. Cyclin B1-mVenus Dynamics during a Complete Cell Cycle, Related to Figure 5 — Representative quantification of mRuby-PCNA and Cyclin B1-mVenus dynamics from mitosis to mitosis. [file mmc4.jpg]

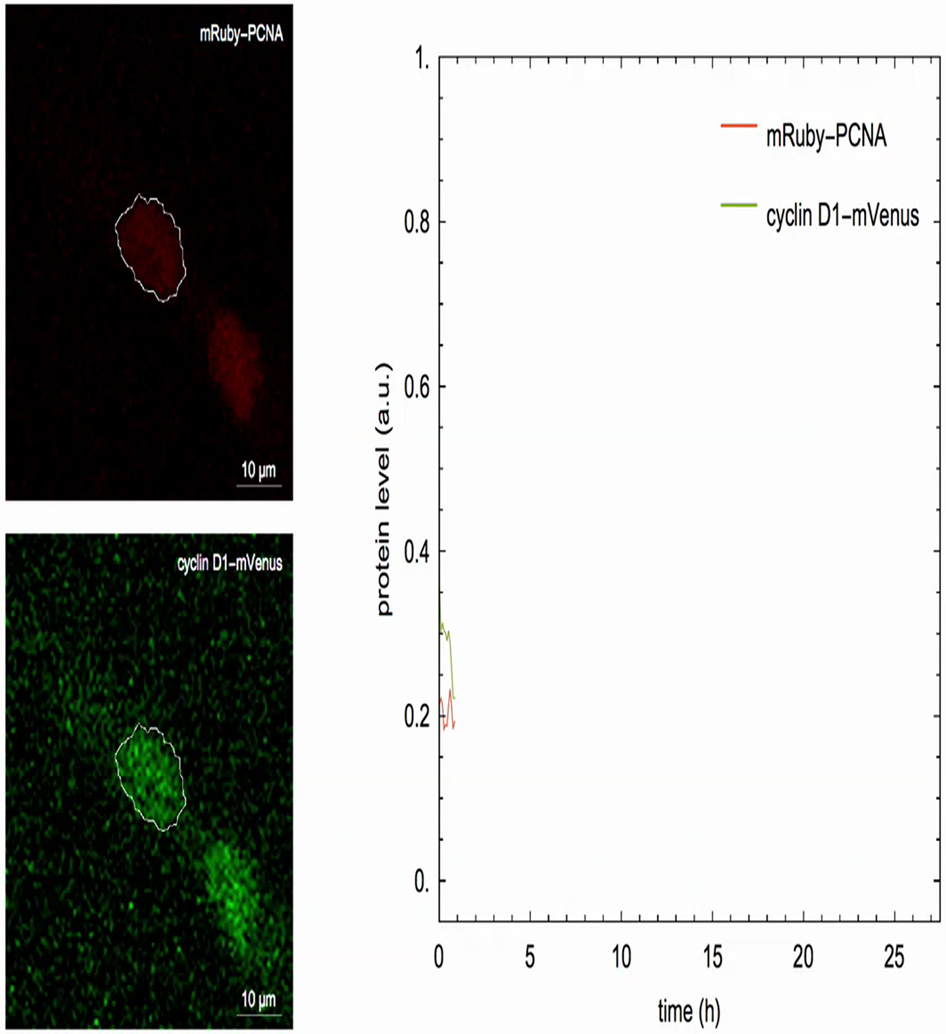

Supplement: Movie S4. Cyclin D1-mVenus Dynamics during a Complete Cell Cycle, Related to Figure 5 — Representative quantification of mRuby-PCNA and Cyclin D1-mVenus dynamics from mitosis to mitosis. [file mmc5.jpg]

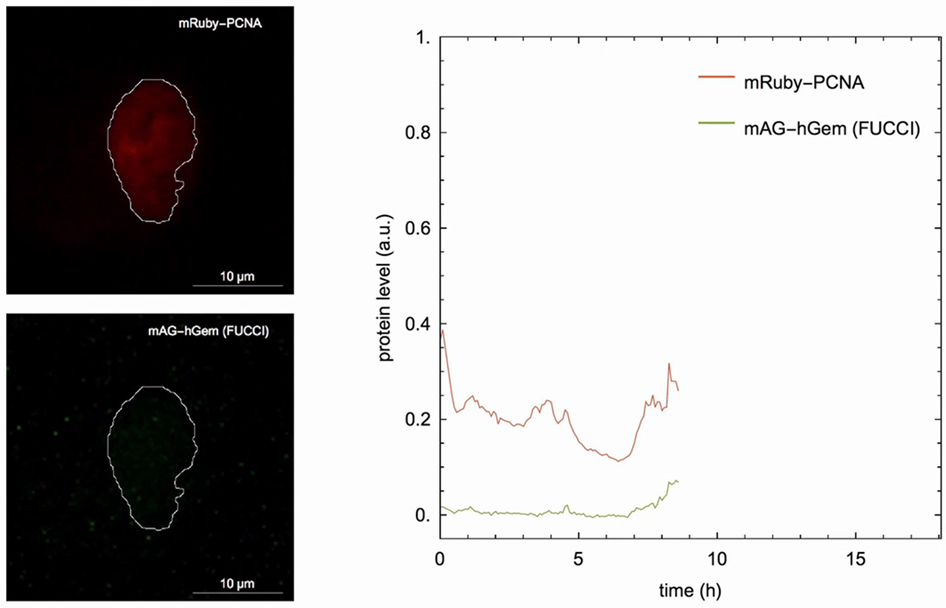

Supplement: Movie S5. mAG-hGem and mRuby-PCNA Dynamics during a Complete Cell Cycle, Related to Figure S2 — Representative quantification of mRuby-PCNA and mAG-hGem (FUCCI) dynamics from mitosis to mitosis. [file mmc6.jpg]
